# Functional and Structural Characterization of a Novel HLA-DRB1*04:01-Restricted α-Enolase T Cell Epitope in Rheumatoid Arthritis

**DOI:** 10.3389/fimmu.2016.00494

**Published:** 2016-11-14

**Authors:** Christina Gerstner, Anatoly Dubnovitsky, Charlotta Sandin, Genadiy Kozhukh, Hannes Uchtenhagen, Eddie A. James, Johan Rönnelid, Anders Jimmy Ytterberg, Jennifer Pieper, Evan Reed, Karolina Tandre, Mary Rieck, Roman A. Zubarev, Lars Rönnblom, Tatyana Sandalova, Jane H. Buckner, Adnane Achour, Vivianne Malmström

**Affiliations:** ^1^Rheumatology Unit, Department of Medicine Solna, Center for Molecular Medicine, Karolinska Institutet, Karolinska University Hospital, Stockholm, Sweden; ^2^Neuroimmunology Unit, Department of Clinical Neurosciences, Center for Molecular Medicine, Karolinska Institutet, Stockholm, Sweden; ^3^Science for Life Laboratory, Department of Medicine Solna, Karolinska Institutet, Stockholm, Sweden; ^4^Translational Research program, BRI at Virginia Mason, Seattle, WA, USA; ^5^Tetramer Core, BRI at Virginia Mason, Seattle, WA, USA; ^6^Department of Immunology, Genetics and Pathology, Uppsala University, Uppsala, Sweden; ^7^Department of Medical Biochemistry and Biophysics, Karolinska Institutet, Stockholm, Sweden; ^8^Science for Life Laboratory, Department of Medical Sciences, Rheumatology, Uppsala University, Uppsala, Sweden; ^9^Department of Infectious Diseases, Karolinska University Hospital Solna, Stockholm, Sweden

**Keywords:** rheumatoid arthritis, HLA-DR4/α-enolase, neo-antigen, CD4^+^ T cell, autoimmunity, cytokines, crystal structures

## Abstract

Antibodies to citrullinated proteins, common in rheumatoid arthritis (RA) patients, are strongly associated to a specific set of HLA-DR alleles including HLA-DRB1*04:01, *04:04, and *01:01. Here, we first demonstrate that autoantibody levels toward the dominant citrullinated B cell epitope from α-enolase are significantly elevated in HLA-DRB1*04:01-positive RA patients. Furthermore, we identified α-enolase-derived T cell epitopes and demonstrated that native and citrullinated versions of several peptides bind with different affinities to HLA-DRB1*04:01, *04:04, and *01:01. The citrulline residues in the eight identified peptides are distributed throughout the entire length of the presented epitopes and more specifically, localized at peptide positions p-2, p2, p4, p6, p7, p10, and p11. Importantly, in contrast to its native version peptide 26 (TSKGLF**R**AAVPSGAS), the HLA-DRB1*04:01-restricted citrullinated peptide Cit26 (TSKGLF**Cit**AAVPSGAS) elicited significant functional T cell responses in primary cells from RA patients. Comparative analysis of the crystal structures of HLA-DRB1*04:01 in complex with peptide 26 or Cit26 demonstrated that the posttranslational modification did not alter the conformation of the peptide. And since citrullination is the only structural difference between the two complexes, this indicates that the neo-antigen Cit26 is recognized by T cells with high specificity to the citrulline residue.

## Introduction

Rheumatoid arthritis (RA) is a chronic inflammatory disease affecting approximately 0.5–1% of the population worldwide (1). Ever since the discovery of the genetic association of RA to a subset of HLA-DR alleles (2), CD4^+^ T cells have been considered as classical effector cells in RA. Furthermore, the importance of CD4^+^ T cell effector functions in RA pathogenesis is corroborated both by therapeutic interventions affecting adaptive immune responses (3, 4) and by multiple studies of polyclonal T cell cytokine production (5, 6). The identification of RA-associated antibody responses to citrullinated proteins and the genetic link established between the presence of such autoantibodies and certain HLA class II molecules (7) has directed the investigation of novel autoantigens, and thereby promoted a renaissance in CD4^+^ T cells studies, as T helper cells would be assumed to govern IgG responses (8). Indeed, the existence of T cell epitopes derived from a range of citrullinated RA-associated autoantigens has recently been demonstrated (9–11). A detailed understanding of when, where, and how tolerance is broken and citrullination-mediated autoimmunity develops may aid toward future preventive strategies.

Still all too little is known about the nature of HLA-DR-restricted native and posttranslationally modified (PTM) T cell self-epitopes that are involved in the development of RA. The main established association between HLA class II molecules and RA has been correlated to the polymorphic region of the β-chain comprising amino acid residues 70–74 (12) that line the MHC-II pocket close to peptide anchor residue p4 and that is shared between HLA-DRB1*04:01, *04:04, and *01 alleles (Figure S1A in Supplementary Material). More recently the importance of residues 11 and 13, localized at the bottom of MHC-II pockets P4 and P6, respectively, for initiation of RA has also been described (13). Both uncharged as well as positively charged residues at positions 13 and 71, respectively, have been suggested to direct the nature of peptide anchor residues that are accommodated within the P4 pocket of the antigen-binding groove of these MHC-II molecules. To our knowledge, only one study has hitherto provided insights in the structural features of citrullinated peptides in complex with HLA-DRB1*04:01, *04:02, and *04:04 (14). This study demonstrated that citrullinated vimentin- or aggrecan-derived peptide residues bind to different MHC class II alleles at peptide positions p2, p4, p9, and/or p11 in a classical manner. In all cases, the citrullinated residue at position p4 could be accommodated within the positively charged pocket of the HLA-DRB1*04:01 and *04:04 alleles, and emerged slightly from the peptide-binding cleft in order to interact with presumptive T cell receptors (TCRs). In contrast, the corresponding negatively charged P4 pocket in the RA-resistant DRB1*04:02 allele disallowed binding of citrullinated epitopes. Thus citrullination of different peptides resulted in the formation of neo-antigens.

In the present study, we focused on citrullinated α-enolase, one of the candidate antigens toward which a large number of RA patients develop autoantibodies. The main B cell epitope in α-enolase, denoted CEP-1, resides between amino acids 5 and 21 (15). Importantly, CEP-1 autoantibodies are highly associated with the shared epitope (SE) HLA-DR alleles (16), including HLA-DRB1*01, *04:01, and *04:04, which are the most common RA-associated alleles in the patient cohort analyzed within this study. An unbiased screening of partially overlapping 15 amino acid long peptides, covering the entire α-enolase protein, allowed us to identify a set of potential T cell epitopes that bind to these three RA-associated-HLA-DR alleles. We demonstrate that T cell responses were significantly enhanced toward citrullinated epitopes in HLA-DRB1*04:01-positive RA patients compared to *01 and *04:04 patients. The most striking responses were established for peptide 26, where only the citrullinated version Cit26 elicited statistically significant T cell responses. We assessed the structural basis underlying these functional differences by determining and comparing the crystal structures of HLA-DRB1*04:01 in complex with the native and citrullinated versions of peptide 26. Our results reveal that the posttranslational modification does not alter the conformation of the citrullinated peptide Cit26 compared to the native peptide 26. The side chain of the citrulline residue at position p2 in Cit26 projects toward the solvent, fully available for interactions with TCRs. This single significant difference between the two complexes suggests that the citrullinated neo-antigen Cit26 may bypass T cell tolerance and provoke undesired responses from T cells with high and possibly unique specificity to the citrulline residue.

## Materials and Methods

### Autoantibody Detection in Serum from a Large Cohort of RA Patients

A total of 2934 RA patients from the Epidemiological investigations in rheumatoid arthritis (EIRA) cohort (17) were investigated with the Phadia ISAC microarray (18) and analyzed for antibodies against the immunodominant α-enolase peptide CEP-1. After exclusion of samples reacting with the chip background or with streptavidin, technically unobjectionable results were obtained from 2858 RA patients with information on HLA-DRB1 alleles. Of these patients, 1396 had one single HLA-DRB1 SE allele with 426 being HLA-DRB1*01, 422 *04:01, and 134 *04:04. The cutoff was set at the 98th percentile for a control group consisting of 578 EIRA healthy controls.

### Patients and Cell Samples

Twenty-six HLA-DRB1*04:01, HLA-DRB1*04:04, and HLA-DRB1*01 RA patients were included in this study (Table S1 in Supplementary Material). All subjects were recruited under the auspices of the Karolinska University Hospital/Karolinska Institutet Arthritis Research Program. Informed consent was obtained from all subjects under protocols approved by the Karolinska Hospital Ethical Review Board. Three HLA-DRB1*04:01-positive blood donors were recruited from the Uppsala Bioresource as healthy controls for this study. Peripheral blood mononuclear cells (PBMCs) were obtained from heparinized blood by centrifugation over Ficoll-Hypaque (GE Healthcare) gradients. PBMCs were cryopreserved in liquid nitrogen in 10% DMSO and 90% heat-inactivated fetal bovine serum.

### Mass Spectrometry Analyses of Citrullinated Recombinant α-Enolase

Recombinant human α-enolase was citrullinated for 2 h at 50°C at a concentration of 1 mg/ml in PAD buffer (100 mM Tris, 10 mM CaCl_2_, 5 mM DTT, pH 7.6) using 2 U/mg protein of rabbit skeletal muscle PAD2 enzyme (Sigma-Aldrich). The reaction was stopped by the addition of EDTA to a concentration of 20 mM, followed by extensive dialysis to calcium-free PBS. Also, 10 μg of the citrullinated enolase were reduced, alkylated, and digested in-solution as previously described (19). After zip tipping (Merck Millipore Ltd., Republic of Ireland), an amount corresponding to 2 pmol prior precipitation and digestion was separated using on-line nLC–MS/MS (RP C18) and analyzed on a Q Exactive MS (Thermo Fisher Scientific, Germany). A 40-min gradient of buffer A and B (A: 1% formic acid in water; B: 1% formic acid in acetonitrile) was used for the separation: 5–30% B for 35 min, followed by 30–95% B for 5 min. The flow rate was 300 nl/min. Mass lists were extracted using Raw2MGF v2.1.3 (20) and used to search a concatenated version of the SwissProt database (2013/4) using the Mascot search engine v2.3.02 (Matrix Science Ltd., London, UK). The following parameters were used for the database searching: tryptic digestion (with a maximum of two miscleavages); carbamidomethylation (C) as fixed modification; oxidation (M), pyroglutamate (Q), deamidation (N/Q), and citrullination (R) as variable modifications; 10 ppm as precursor tolerance and 0.1 Da as fragment tolerance. Spectra identifying citrullinated peptides were validated manually by verifying that the precursor mass was correctly assigned, and that the modified site was consistent with observed mass shifts in the fragment ions.

### HLA-DRB1 Peptide-Binding Assays

Stepwise binding analysis of 15-mer peptides derived from both native and citrullinated α-enolase to HLA-DRB1*01, *04:01, and *04:04 was performed using the ProImmune Class II Reveal assay (ProImmune, Oxford, UK). Relevant peptides were thereafter synthesized by GenScript (Piscataway, NJ, USA) and used for competition peptide-binding assays. Epitopes were incubated in increasing concentrations in the presence of plate-bound HLA-DRB1*01, *04:01, or *04:04 and 0.02 μM of the biotinylated competitor epitopes HA_306–318_ (PKYVKQNTLKLAT) for HLA-DRB1*04:01 and *01:01, and GAD65_270–285_ (LPRLIAFTSEHSHFS) for HLA-DRB1*04:04 (21). Finally, peptide-binding affinity to the three different HLA alleles was assessed in direct binding assays in which *N*-terminally biotinylated epitopes (GenScript, Piscataway, NJ, USA) were incubated at concentrations from 5 to 0.05 μM with plate-bound relevant HLA-DRB1 molecules in the absence of a competitor peptide (22). The inflection point of the binding curve of a known binding epitope was used as a cutoff to separate weakly binding epitopes from background.

### Functional Cellular Assays

Functional T cell assays were performed as previously described (9). PBMCs were cultured for 5 days in the presence of 20 μg/ml peptides at a total cell concentration of 1 × 10^6^ cells/well in flat bottom 96-well plates in RPMI 1640 supplemented with 2 mM l-glutamine, 100 U/ml penicillin, 100 μg/ml streptomycin, 10 mM HEPES, and 10% pooled human serum. PBMCs were restimulated with peptides and anti-CD28 (BioLegend) on day 5 for 6 h with 5 μg/ml Brefeldin A (Sigma-Aldrich) being added for the last 4 h. Restimulated cells were treated with LIVE/DEAD^®^ Fixable Green Dead Cell Stain (Invitrogen) and thereafter stained for surface expression of CD3 (BioLegend), CD4, and CD14 (BD Biosciences). Cells were then stained intracellularly for IFN-γ and IL-17A (BioLegend) as well as CD154 (CD40L) (BD Biosciences) expression using the Cytofix/Cytoperm fixation and permeabilization solution kit (BD Biosciences). Samples were run on a CyAn™ ADP Analyzer (Beckman Coulter), and data were analyzed using FlowJo software, version 7.5.1 or higher (Tree Star). The gating strategy is depicted in Figure S2 in Supplementary Material. In some experiments, blocking antibodies for HLA-DR (clone L243), -DP (clone B7/21), and -DQ (clone SPLV3), all obtained from the Tetramer Core Facility (BRI, Seattle, WA, USA), were added to the cultures.

### Cytokine Detection in Supernatants

A bead-based multiplex cytokine assay was custom-designed by Invitrogen for simultaneous detection of IL-10, IL-13, IL-17A, IL-17F, TNF-α, and IFN-γ on a Luminex platform. Supernatants were collected at day 5 and stored at −80°C until used. Supernatants from RA patients were analyzed using the Luminex 100 system according to the manufacturer’s instructions.

### Production and Isolation of HLA-DRB1*04:01 in Complex with Peptides 26 and Cit26

Peptides 26 and Cit26 were purchased at >95% purity from GenScript (Piscataway, NJ, USA). The extracellular domains of the HLA-DRB1*04:01 α- and β-chains with an acidic and basic leucine zipper, respectively, as well as a C-terminal hexahistidine tag were expressed separately in *E. coli* BL21 (DE3) STAR cells (Novagen). Inclusion bodies, dissolved in 8M urea, 50 mM Tris–HCl (pH 8) were purified on a HiTrapQ HP anion exchange column (GE Healthcare). The purified α- and β-chains were diluted to a final concentration of 2 mg/ml each in a refolding solution containing 50 mM Tris–Citrate buffer pH 7.5, 25% (w/v) glycerol, 0.01% Pluriol F68, and 5 μM peptide. HLA-DRB1*04:01 in complex with either peptides 26 or Cit26, obtained after 72 h *in vitro* room temperature refolding, were concentrated using a 10-kDa cutoff Vivaspin Turbo 15 (Sartorius) and thereafter dialyzed against 25 mM Tris–HCl, pH 8.0. Both complexes were isolated using anion exchange on a Mono Q5/50 column followed by size-exclusion chromatography on a Superdex 200 column (GE Healthcare). The monomeric MHC-II/peptide complexes were concentrated to 1 mg/ml, subjected to thrombin cleavage in order to remove the leucine zippers and further purified using a HiTrap Chelating HP column (GE Healthcare). The flow-through was pooled, concentrated using a 10-kDa cutoff Vivaspin Turbo 15 (Sartorius), and further purified on a Superdex 200 column. Fractions containing monomeric MHC-II/peptide complexes were concentrated using a 10-kDa cutoff Vivaspin Turbo 4 (Sartorius) to 10 mg/ml.

### Crystallization and Structure Determination of the HLA-DRB1*04:01/26 and HLA-DRB1*04:01/Cit26 Complexes

Crystals of the HLA-DRB1*04:01/26 and HLA-DRB1*04:01/Cit26 complexes were obtained using the hanging-drop vapor diffusion method at 20°C. Protein solution and a mother liquor of 100 mM sodium malonate, pH 4.0, 12–18% (vol/vol) PEG3350 were mixed at a 1:1 ratio and equilibrated against 1 ml of mother solution. Rod-like crystals typically grew within 2–6 days. Crystals were flash frozen in liquid nitrogen after soaking in 35% PEG 3350. X-ray diffraction data for the HLA-DRB1*04:01/Cit26 complex were collected at the ID23-1, European Synchrotron Research Facility, and for the HLA-DRB1*04:01/26 complex at the beamline BL14-1, Helmholtz-Zentrum Berlin, and processed using the programs XDS (23, 24) and XDSAPP (25). The crystal structures were determined by molecular replacement using the program Phaser (26) and a HLA-DRB1*04:01/peptide complex (PDB code 4MCY) (14) with the peptide omitted and subsequently refined using Refmac5 and iterations of manual refinement using Coot (27). The crystal structures were validated using MOLPROBITY (Table 3) (28). The coordinates and structural factors of the crystal structures of the HLA-DRB1*04:01 in complex with peptides 26 and Cit26 have been deposited to the Protein Data Bank under accession codes 5LAX and 5JLZ, respectively.

### Statistical Analysis

Statistical analyses were performed using Prism 6 software (Graph Pad, San Diego, CA, USA). If not stated otherwise, mean with SD is depicted. Detailed information on statistical tests can be found in the figure legends. *P* values less than 0.05 were considered significant and depicted with an asterisk.

## Results

### Autoantibody Levels toward Citrullinated α-Enolase Are Significantly Enhanced in HLA-DRB1*04:01-Positive Compared to HLA-DRB1*01- or *04:04-Positive RA Patients

**Figure 1:**
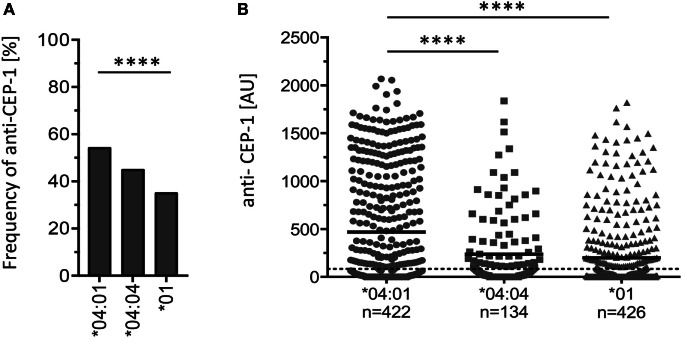
The amount of autoantibodies toward CEP-1 in citrullinated α-enolase is significantly enhanced in HLA-DRB1*04:01-positive subjects compared to *01- and *04:04-positive subjects. **(A)** Frequency of RA patients positive for anti-CEP-1 antibodies among HLA-DRB1*04:01-positive (*n* = 228 out of 422), *04:04-positive (60/134), and *01-positive individuals (149/426). The cutoff for the ELISA assay was set at the 98th percentile based on a control group consisting of 578 EIRA healthy controls. For overall comparison of the frequency of anti-CEP-1 positivity in patients carrying a certain genotype, we used Pearson’s chi-squared test. Four asterisks correspond to 0.0001. **(B)** Levels of anti-CEP-1 antibodies in HLA-DRB1*04:01-, *04:04-, and *01-positive RA patients. Cutoff is set at 83.08 AU. Unpaired Student’s *t*-test was used to compare anti-CEP-1 antibody levels. Four asterisks correspond to 0.0001.

We hypothesized that different RA-associated HLA-DR alleles could bias citrulline-based antibody-mediated autoimmunity and therefore first investigated the prevalence of autoantibodies toward the major B cell epitope CEP-1 on citrullinated α-enolase. Based on the EIRA cohort (17), 982 RA patients who carried one copy of either HLA-DRB1*04:01, *04:04, or *01 and with available CEP-1 data were identified. This initial analysis revealed significantly higher frequencies and levels of antibodies specific to CEP-1 in HLA-DRB1*04:01 patients compared to HLA-DRB1*04:04 and *01 patients (Figure 1).

### Identification of Novel HLA-DR-Restricted α-Enolase-Derived T Cell Epitopes

We first performed an unbiased binding screen using both native and citrullinated 15-mer peptides overlapping by five amino acid residues, covering the entire protein (data not shown). This assay, the so-called ProImmune Class II Reveal assay (ProImmune, Oxford, UK), provided initial indications for binding to HLA-DRB1*01, *04:01, and *04:04 (Table S2 in Supplementary Material). The peptides with the highest scores were selected for further analysis (Table 1). The crystal structure of human α-enolase homodimer (PDB entry 3B97) (30) allowed us to evaluate the propensity of all arginine residues to be citrullinated. All the peptides identified through the first screening were exposed to the solvent (Figure S1B in Supplementary Material). Furthermore, mass spectrometry analyses of *in vitro* citrullinated recombinant α-enolase protein revealed that at least five of the identified epitopes (peptides 1, 11, 56, 171, and 420) were citrullinated (Table 1), while the native forms of the three remaining peptides (26, 241, and 326) could not be identified and therefore their propensity for citrullination could not be assessed.

**Table 1:** Sequence of candidate peptides, aligned according to the predicted position in the groove of HLA-DRB1*04:01, *in vitro* citrullination.

Peptide ID	Amino acid numbers	Sequence/pockets 1 4 6 9	*In vitro* citrullination
1/Cit1	1–15	MSI LKIHA**R**EIF DS**R**	9, 15
11/Cit11	11–25	I FDS**R**GNPTV EDVLF	15
26/Cit26	26–40	TSKGL F**R**AAVPSGA S	n.d.
56/Cit56	56–70	**R**Y MGKGVSKAV EHIN	56
171/Cit171	171–185	LP VGAANF**R**EA M**R**IG	183
241/Cit241	241–255	VIG MDVAASEFF **R**SG	n.d.
326/Cit326	326–340	K**R** IAKAVNEKS CNCL	n.d.
420/Cit420	420–434	KAK FAG**R**NF**R**NP LAK	426, 429

*n.d., not detected, i.e., peptides were not found, arg vs. cit status could thus not be determined*.

The sequences of all the identified peptides were aligned according to the HLA-DR pocket preferences at P1, P4, P6, and P9, indicating that citrulline residues can be positioned in different pockets within the clefts of HLA-DRB1*04:01, *04:04, and *01, or alternatively can protrude toward TCRs (Table 1). It should be noted that only peptides 11 and 420 comprised arginine/citrulline residues that could act as anchoring positions within pocket P4. The arginine/citrulline residues in peptides 1, 171, and 420 may either interact with pockets P6 or P7, or protrude out of the HLA cleft. In all the other cases, the arginine/citrulline residues localized at peptide positions p-2, p-1, p2, p10, p11, and p12 are predicted to protrude out of the cleft, readily available for interactions with TCRs (Table 1).

### Native and Citrullinated α-Enolase-Derived Peptides Bind with Different Affinities to HLA-DRB1*04:01, *04:04, and *01:01

**Figure 2:**
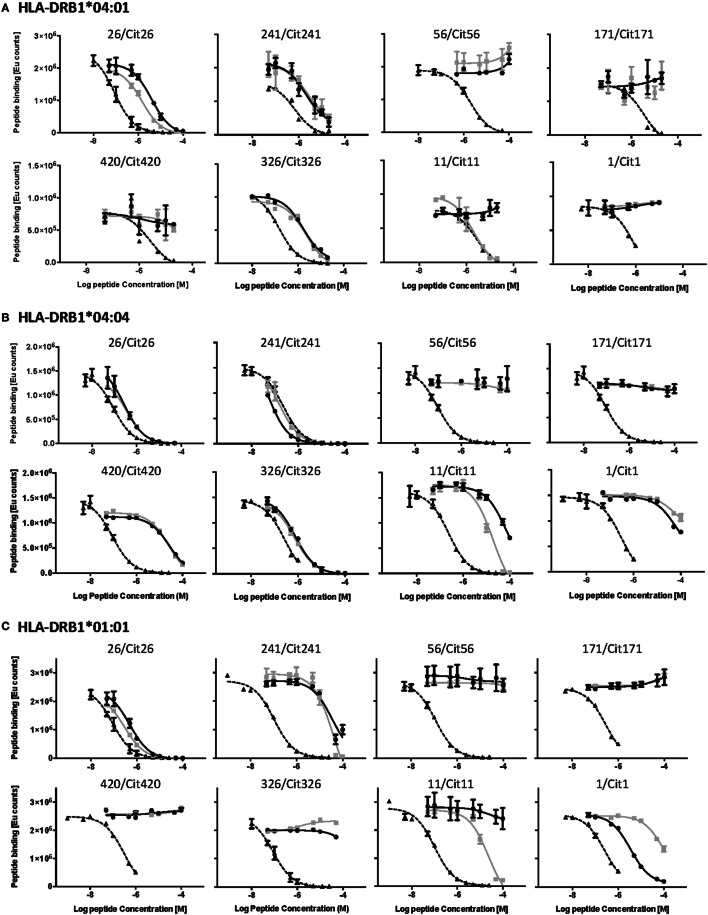
Competition binding assays demonstrate the differential capacity of native and citrullinated α-enolase-derived peptides to bind to HLA-DRB1*04:01, *04:04, and *01:01. The upper, middle, and lower panels present the results from the competition binding assays for all the eight identified α-enolase-derived peptides to **(A)** HLA-DRB1*04:01, **(B)** HLA-DRB1*04:04, and **(C)** HLA-DRB1*01:01, respectively. The curves for the reference peptide, native, and citrullinated α-enolase peptides are displayed in dashed, gray, and black lines, respectively. All plots are arranged according to the order of appearance in Table 2.

The HLA-DR binding capacity of the identified peptides was assessed through *in vitro* competition binding assays in which native and citrullinated candidate epitopes were tested for their capacity to displace an established reference peptide (Figure 2; Table 2). We decided to make use of HLA-DRB1*01:01 as a representative model for HLA-DRB1*01. Our analyses revealed that both the native and citrullinated versions of peptides 26 and 241 were able to efficiently compete with the reference peptide in the context of HLA-DRB1*04:01, *04:04, and *01:01. In contrast, neither native nor citrullinated peptides 56 and 171 could displace the reference peptide in any of the three MHC class II molecules. Furthermore, both versions of peptide 420 were unable to compete with the reference peptide in HLA-DRB1*04:01 and *01:01 but could weakly displace the HLA-DRB1*04:04 reference peptide.

**Table 2:** **Results of the competition and direct binding assays**.

	IC_50_ (μM)		
Peptide	*04:01	*04:04	*01:01
26	4.0	0.5	1.4
cit26	1.5	0.5	1.9
241	2	0.5	35
cit241	2.5	0.2	42
56	–	–	–
cit56	–	–	–
171	–	–	–
cit171	–	>50	–
420	>50	25	>50
cit420	>50	28	>50
326	1.7^a^	0.7	>50
cit326	1.9^a^	0.7	>50
11	>50^a^	>50	–
cit11	2.2^a^	1.7	25
1	–	>50	3.5
cit1	–	>50	>50

*>50, weak binding confirmed in direct binding assay*.

*^a^Published in Ref. (29)*.

*–, No binding detected*.

Clear differences were established for the capacity of 1/Cit1, 11/Cit11, and 326/Cit326 to compete with reference peptides in the context of either all three or only one of the MHC class II molecules. While both 326 and Cit326 efficiently displaced the reference peptide in HLA-DRB1*04:01 and *04:04, they were not able to do so with HLA-DRB1*01:01. Interestingly, citrullination of peptide 11 significantly enhanced competition efficiency in the context of all three MHC alleles. In contrast, the native version could not compete in HLA-DRB1*01:01 and *04:01 and was able to only weakly compete the reference peptide in HLA-DRB1*04:04. Finally, a converse situation was observed for peptide 1 with HLA-DRB1*01:01, where citrullination significantly reduced the capacity of Cit1 to compete. Neither native nor citrullinated version of this peptide demonstrated significant competition with the reference peptide in the context of HLA-DRB1*04:01 and *04:04.

**Figure 3:**
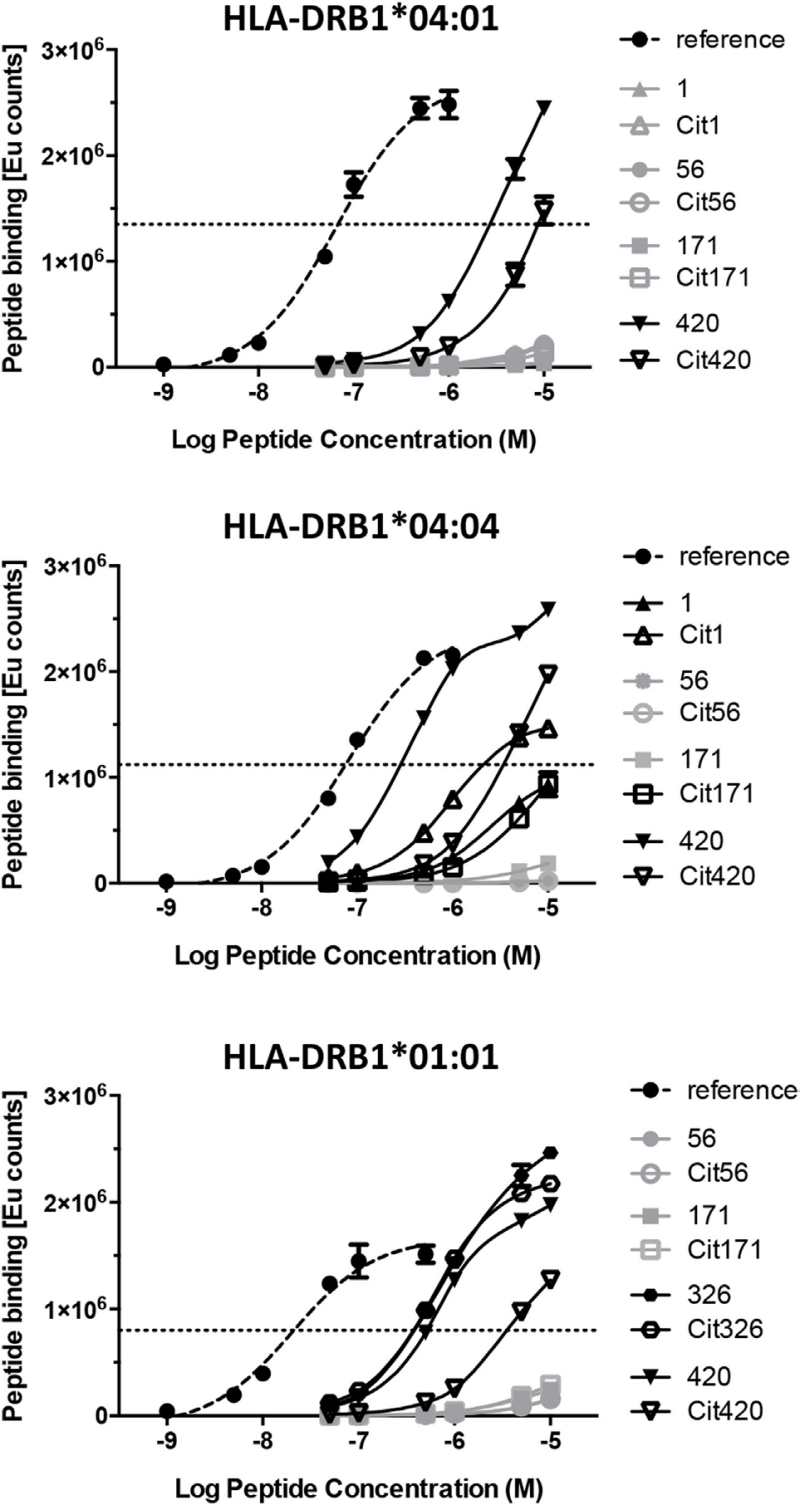
**Direct binding assays reveal the capacity of specific α-enolase-derived peptides to bind to the three different MHC alleles**. Direct binding assays were performed for peptide pairs that did not bind or bound poorly to the three different MHC alleles in the competition binding assays. The point of inflection in the curve for the reference peptide was defined as the binding threshold reference point. Thus, any peptide that did not reach or barely passed this level was assessed as non-binding. Curves for the reference peptide, the non-binding, and binding peptides are displayed in dashed, gray, and black lines, respectively.

It is probable that the binding thresholds within our competition assays exceed the requirements for weakly binding peptides that still can be relevant functionally. Indeed, an example of a peptide that appears negative in MHC binding assays, but still provides functional T cell responses, has been previously identified (22). We therefore assessed the direct binding of biotinylated versions of all the native and citrullinated peptides that did not or did only very weakly displace reference peptides in our competition assays, to plate-bound HLA-DRB1*04:01, *04:04, and *01:01 (Figure 3). Specific *N*-terminal biotinylation of peptides *via* ε-aminocaproic acid linker prevents modification of lysine residues within the peptides and is thus not expected to affect peptide–MHC interaction. No significant binding could be confirmed for native or citrullinated versions of peptides 56 and 171. Conversely, although the native and citrullinated versions of peptide 326 could not displace the reference peptide in HLA-DRB1*01:01, both 326 and Cit326 bound well to this MHC allele in direct binding assays (Figure 3; Table 2). Also in contrast to the competition assay results, both versions of peptide 420 bound well to all three MHC class II alleles, however, always with a significantly reduced binding capacity for the citrullinated version compared to the native form. Finally, the direct binding assays confirmed that both forms of peptide 1 do not bind to HLA-DRB1*04:01 and bind only weakly to HLA-DRB1*01:01 (Figure 3; Table 2).

### In Contrast to Native Versions, Several HLA-DRB1*04:01-Restricted Citrullinated Peptides Elicit Functional T Cell Responses in Primary Cells from RA Patients

**Figure 4:**
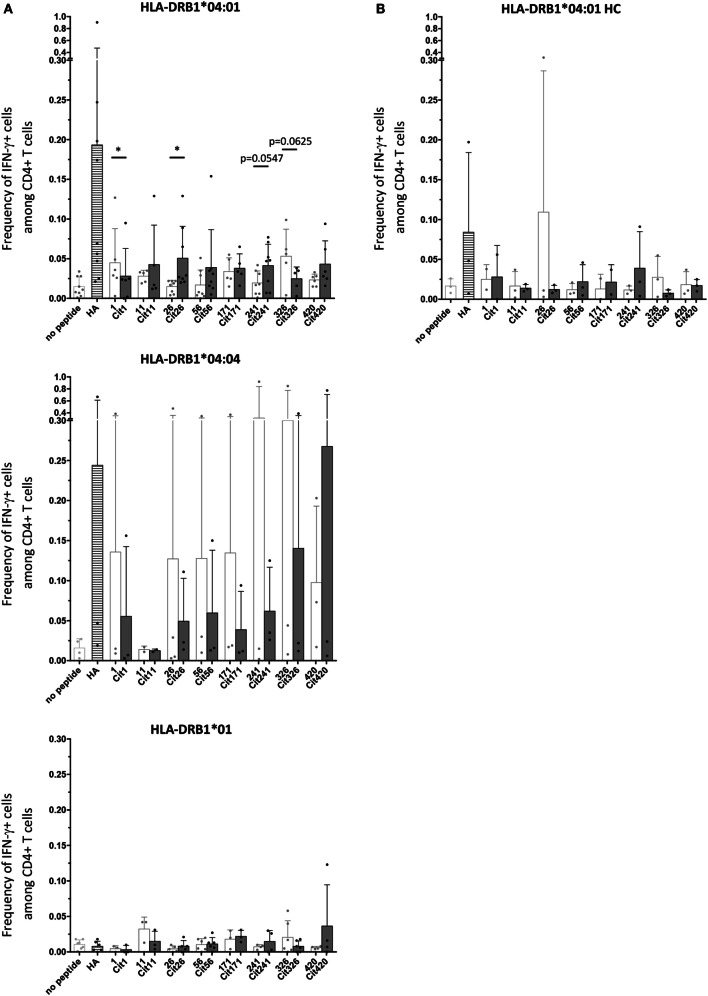
Cytokine production in cultured primary cells from HLA-DRB1*04:01-positive RA patients is significantly increased to the citrullinated peptide Cit26 compared to the native peptide 26. **(A)** Summary of intracellular flow cytometry stainings for IFN-γ following 5 days of culture of RA patient samples with or without peptide stimulation. Single patient values are indicated by dots. The mean with SD is depicted for unstimulated cultures (light gray) as well as cultures stimulated with the positive control peptide HA (striped bar) and the eight peptide pairs (light gray bars: native versions; dark gray bars: citrullinated versions), respectively. Statistical analysis was performed using Wilcoxon signed rank test when comparing levels of IFN-γ production in PBMCs stimulated with native or citrullinated peptides. Chauvenet’s criterion was used to exclude possible experimental outliers; however, all data points were calculated to be in range and thus kept in the analysis. Number of patient samples included for HLA-DRB1*04:01 is nine with eight of these used for peptide pairs 26/Cit26, 56/Cit56, and 241/Cit241, six for peptide pairs 1/Cit1 and 420/Cit420, and five for 11/Cit11, 171/Cit171, and 326/Cit326. For HLA-DRB1*04:04, we used five patient samples in total, with peptide pairs 1/Cit1, 56/Cit56, 171/Cit171, 241/Cit241, 326/Cit326, and 420/Cit420 being tested in three of those and peptide pair 11/Cit11 in two. Three HLA-DRB1*01 patient samples were used for all peptide pairs except for 1/Cit1 and 171/Cit171, where only two samples were tested. **(B)** Intracellular flow cytometry stainings for IFN-γ after 5 days of culture of three HLA-DRB1*04:01 healthy control samples with or without peptide stimulation do not show the same level and breadth of response to citrullinated peptides as HLA-DRB1*04:01-positive RA patients. Besides single values, the mean with SD is depicted for unstimulated cultures (light gray) as well as cultures stimulated with the positive control peptide HA (striped bar) and the eight peptide pairs (light gray bars: native versions; dark gray bars: citrullinated versions), respectively. Number of samples tested was three for all the peptide pairs except for peptide pair 1/Cit1 and 171/Cit171 where only two samples were tested.

Primary cells from HLA-typed RA patients (Table S1 in Supplementary Material) were screened for T cell activation, assessed by CD40L upregulation as well as intracellular cytokine staining (ICS), following *in vitro* stimulation with native or citrullinated variants of the identified peptides. As expected, cytokine production is largely confined to the CD40L^+^ T cell subset (Figure S2 in Supplementary Material). IL-17A and/or IFN-γ production were observed in conjunction with a set of native and/or citrullinated α-enolase peptide stimulations, especially in the context of HLA-DRB1*04:01 (Figure 4; Figure S3 in Supplementary Material). Notably, T cell responses in HLA-DRB1*04:01-positive patient samples were significantly increased toward the citrullinated peptide Cit26 compared to its native counterpart (Figure 4A). Although not statistically significant, similar trends were observed for peptides Cit11, Cit56, Cit241, and Cit420 compared to their native counterparts. A similar trend was also observed in HLA-DRB1*01-positive patient samples with peptide 420. In contrast, the citrullination of peptides 1 and 326 decreased T cell responses in HLA-DRB1*04:01-positive patients (Figure 4A).

Next, we assessed T cell responses elicited by the identified native or citrullinated peptides in HLA-DRB1*04:01-positive healthy controls. In contrast to RA patients, most T cell responses were comparable to the unstimulated threshold value. Thus, our results indicate that the T cell responses toward HLA-DRB1*04:01-restricted citrullinated peptides are more robust and pro-inflammatory in RA patients compared to healthy controls (Figure 4; Figure S3 in Supplementary Material). Furthermore, to ensure that the observed peptide responses were indeed restricted by HLA-DR alleles and not influenced by accompanying HLA-DP or HLA-DQ alleles, we also performed stimulation experiments where blocking antibodies toward the different HLA class alleles were included. In contrast to HLA-DP- or HLA-DQ-blocking antibodies, only HLA-DR-blocking antibodies were able to ablate responses to the stimulatory peptides (Figure S4 in Supplementary Material).

In conclusion, the citrullinated versions of peptides 26, 56, and 241 result in enhanced T cell responses in HLA-DRB1*04:01-positive patients. In line with these findings, analysis of a broad cytokine panel in supernatants from cultured PBMCs following stimulation with either native or citrullinated peptides also revealed strong responses toward Cit26, Cit56, and Cit241 compared to the native peptide forms (Figure S5 in Supplementary Material).

### Citrullination at Peptide Position 2 Does Not Alter the Conformation of Cit26 Compared to the Native Peptide 26 and Results in the Formation of a Neo-antigen

**Figure 5:**
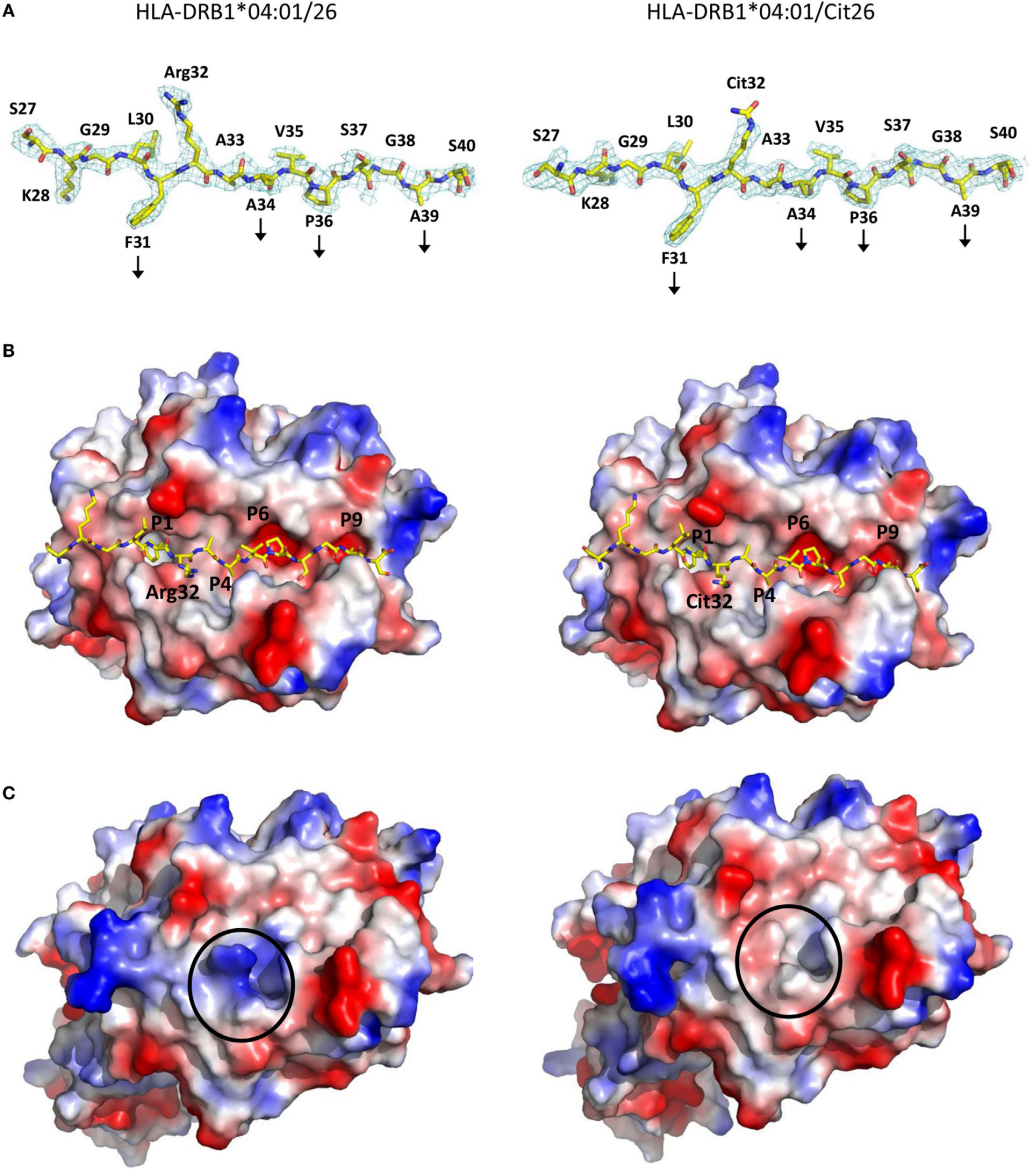
The crystal structures of HLA-DRB1*04:01/26 and HLA-DRB1*04:01/Cit26 reveal how peptide citrullination creates a neo-antigen that may select for a new T cell repertoire. **(A)** The 2Fo-Fc electron density maps of peptides 26 (left) and Cit26 (right) bound to HLA-DRB1*04:01 contoured at 1.0 σ allow for unambiguous positioning of all side chains. The peptides are depicted with their N and C termini to the left and right, respectively, illustrating the main anchor positions by vertical arrows. Residues p31F, p34A, p36P, and p39A are buried in pockets P1, P4, P6, and P9, respectively, within the HLA-DRB1*04:01 peptide-binding cleft. Residues p30L, p32R/Cit, and p35V are accessible for interactions with TCRs. Importantly, both residues p32R and p32Cit in HLA-DRB1*04:01/26 and HLA-DRB1*04:01/Cit26 are extended toward the solvent and may therefore represent a key interaction contact with TCRs. Carbon, nitrogen, and oxygen atoms are in yellow, blue, and red, respectively. **(B)** Peptides 26 (left) and Cit26 (right) are represented as stick models, while HLA-DRB1*04:01 is represented by its surface (viewed from above). Negatively and positively charged regions of the MHC-II surface are given in red and blue, respectively. The pockets permitting binding of different sections of the peptides are displayed. **(C)** Electrostatic potential representation of the surfaces of the HLA-DRB1*04:01/26 and the HLA-DRB1*04:01/Cit26 complexes (left and right, respectively) reveal that the positive charges from the side chain of the arginine residue in peptide 26 are abrogated upon substitution to a citrulline residue in peptide Cit26.

In order to assess the structural bases that underlie stronger T cell responses toward HLA-DRB1*04:01 in complex with the PTM peptide Cit26 compared to the native α-enolase epitope 26 in RA patients, we determined the crystal structures of HLA-DRB1*04:01 in complex peptides 26 and Cit26 at 2.6 and 2.0 Å resolution, respectively (Figure 5; Table 3). Both peptides take prototypic conformations, extending throughout the length of the binding cleft of HLA-DRB1*04:01, using residues p31F, p34A, p36P, and p39A to anchor in pockets P1, P4, P6, and P9, respectively. Comparative analysis of both complexes reveals that all HLA-DRB1*04:01 and the peptide residues keep exactly the same conformations. The only difference between the two crystal structures is the side chain of the citrullinated residue p32Cit in Cit26, which similarly to the arginine residue in HLA-DRB1*04:01/26, protrudes toward the solvent readily available for interactions with TCRs. Thus, citrullination of peptide 26 creates a neo-antigen that could select for an entirely different autoreactive T cell repertoire, with most probably high specificity to the citrulline residue in Cit26.

**Table 3:** Data collection and refinement statistics of the crystal structure of DRB1*04:01 in complex with α-enolase peptide 26–40 with citrulline-32 or arginine-32.

	Citrulline 26–40	Arginine 26–40
**Data collection**		
Beamline	ID23-1, ESRF	BL14-1, BESSY
Wavelength (Å)	0.976	0.918
Space group	P2_1_2_1_2_1_	P2_1_2_1_2_1_
a (Å), b (Å), c (Å)	69.4, 73.8, 145.4	69.2, 73.5, 144.6
Resolution (Å)	47.76–1.99 (2.05–1.99)	48.2–2.6 (2.75–2.6)
No. of observed reflections	274,263	138,347
No. of unique reflections	51,357	23,329
Multiplicity	5.3	5.9
Completeness (%)	99.1 (96.8)	99.6 (98.8)
^a^*R*_meas_ (%)	11.3 (69.8)	21.8 (98.8)
1/σ(I)	9.9 (2.1)	7.8 (1.8)
CC (1/2) (%)	99.7 (84.9)	98.9 (63.7)
Wilson *B*-value (Å^2^)	38.3	38.6
**Refinement statistics**		
Resolution of data (Å)	47.76–1.99	47.56–2.6
^b^*R*_cryst_ (%)	20.6	19.1
^c^*R*_free_ (%)	24.3	27.0
No. of protein atoms	6134	6112
No. of peptide atoms	198	190
Water molecules	366	266
No. of malonate atoms	7	7
**Rmsd from ideal geometry**		
Bond lengths (Å)	0.014	0.013
Bond angles (°)	1.612	1.614
**Ramachandran plot (%)**		
Residues in preferred regions	97.0	96.4
Residues in allowed regions	3.0	3.3
Outliers	0	0.3
Average *B*-value (Å^2^)	46.0	44.0
Protein	47.1	44.8
Peptide	43.2	41.0
Water	43.0	32.3

*Values in parentheses refer to the highest resolution shell*.

*^a^$R_{\text{meas}}=\frac{\sum_{\mathrm{hkl}}\sqrt{\frac{n}{n-1}}\sum_{j=1}^{n}|I_{\mathrm{hkl},j}-\langle I_{\mathrm{hkl}}\rangle |}{\sum_{\mathrm{hkl}}\sum_{j}I_{\mathrm{hkl},j}}$ redundancy independent R-factor (intensities) (28)*.

*^b^*R*_cryst_ = Σ||F_o_| − |F_c_||/Σ|F_o_|, where |F_o_| and |F_c_| are the observed and calculated structure factor amplitudes of a particular reflection, and the summation is over 95% of the reflections in the specified resolution range. The remaining ~5% of the reflections were randomly selected (test set) before the structure refinement and not included in the structure refinement*.

*^c^R_free_ was calculated over these reflections using the same equation as for R_cryst_*.

## Discussion

Using the HLA-DRB1*01:01, *04:01, and *04:04 alleles that are all genetically associated with RA and commonly found in our cohort of RA patients, we initiated this study by demonstrating that the autoantibody levels toward citrullinated α-enolase are significantly increased in HLA-DRB1*04:01-positive RA patients compared to *01 and *04:04 RA patients. These results provided us with a first indication that T cell responses in HLA-DRB1*04:01-positive RA patients could be stronger toward α-enolase-derived peptides compared to *01 and *04:04 RA patients. Covering the entire protein, we identified eight 15-mer peptides that tentatively could be presented by HLA-DR (Table 1). Only two of the identified epitopes comprised an arginine/citrulline residue that occupies the HLA class II pocket P4 (peptides 11/cit11 and 420/Cit420). In all the remaining identified peptides, the arginine/citrulline residues localized at positions p-2, p-1, p2, p10, p11, and p12 were predicted by molecular modeling to protrude out of the cleft, readily available for interactions with TCRs (data not shown). Thus, our results indicate that citrullination can occur on virtually any position in HLA class II-restricted peptides. Peptides able to bind to the RA-associated HLA-DR alleles are candidates for HLA-DR-tetramer approaches where the autoreactive T cell repertoire can be assessed in patient material. Interestingly, we have previously reported the presence of tetramer-positive T cells to two of these α-enolase epitopes (cit11 and cit326) in the context of HLA-DRB1*04:01-positive healthy subjects and RA patients (29).

We could also demonstrate diverse peptide preferences for the three different HLA-DR alleles, and that citrullination can enhance or reduce the binding affinity of peptides to MHC-II molecules. For HLA-DRB1*04:04, the relatively smaller size of the P1 pocket may hinder binding of some of the epitopes favored by HLA-DRB1*04:01. In contrast, pockets P4, P6, and P7 in HLA-DRB1*04:04 comprise different amino acid residues that alter the size (but not the charge) of the pockets (16). Still, we cannot fully explain why the functional bias toward citrullinated peptides was more prominent in the HLA-DRB1*04:01 setting compared to the two other alleles. Recently, it has been demonstrated that the genetic association to amino acids 11, 71, and 74 in the HLA-DRB1 chain correlates with disease severity in HLA-DRB1*04:01- followed by *04:04-positive RA patients (31). Of note, in the present study, we have actively excluded patients carrying more than one copy of the RA-associated HLA-DRB1 alleles.

Native and citrullinated versions of all the identified α-enolase-derived peptides were tested for their capacity to elicit functional T cell responses. Significant citrulline-dependent responses were confirmed only for peptide 26 (TSKGLF**R**AAVPSGAS). Interestingly, both the native and citrullinated versions of peptide 26 bound equally well to both HLA-DRB1*04:04 and HLA-DRB1*01:01. In contrast, a small but significant increase in affinity to HLA-DRB1*04:01 was measured for Cit26 compared to 26. Comparative analyses of the crystal structures of HLA-DRB1*04:01/26 and HLA-DRB1*04:01/Cit26 demonstrated that the only structural difference between the two MHC-II/peptide complexes was the modification from arginine to citrulline at peptide position p2. The structures reveal that both the arginine or the citrulline project readily toward the solvent, with the tip of their side chains localized very close to the central part of the presented peptides and thus fully available for interactions with CDR3s, the most sensitive and specific regions of TCRs. Our structural analysis also indicates that this singular posttranslational modification creates a neo-antigen that may thus select for a repertoire of T cells with high specificity to the citrulline residue in Cit26. Thus, we hypothesize here that T cell subsets that have not been eliminated during negative selection could be selected through citrullination of peptide 26.

Citrulline autoimmunity is a well-recognized feature of ACPA-positive RA. Studies of autoantibodies have made it clear that several modified proteins are recognized. It remains to be established if all these autoantibody responses are dependent on CD4^+^ T cell help. In this context, we are fully aware that α-enolase represents only one of several RA candidate autoantigens that are abundantly expressed in the rheumatic joint (32–35), but our study points to the importance of carefully selecting the studied T cell epitopes in the context of the HLA-DR alleles that are carried by patients in different cohorts and their validation through functional T cell assays. Hopefully, this peptide will be useful for immuno-monitoring studies of RA in conjunction with other validated T cell epitopes.

## Author Contributions

CG, AD, and CS contributed to data generation and analysis and prepared the manuscript. GK, HU, EJ, JR, AY, JP, ER, MR, KT, LR, and RZ contributed to data generation and analysis and reviewed the manuscript. TS and JB contributed to data analysis and reviewed the manuscript. AA and VM conceived the study, contributed to data analysis, and prepared the manuscript.

## Conflict of Interest Statement

The authors declare that the research was conducted in the absence of any commercial or financial relationships that could be construed as a potential conflict of interest.
