# Corrigendum: Functional and Structural Characterization of a Novel HLA-DRB1*04:01-Restricted α-Enolase T Cell Epitope in Rheumatoid Arthritis

**DOI:** 10.3389/fimmu.2017.01236

**Published:** 2017-10-04

**Authors:** Christina Gerstner, Anatoly Dubnovitsky, Charlotta Sandin, Genadiy Kozhukh, Hannes Uchtenhagen, Eddie A. James, Johan Rönnelid, Anders Jimmy Ytterberg, Jennifer Pieper, Evan Reed, Karolina Tandre, Mary Rieck, Roman A. Zubarev, Lars Rönnblom, Tatyana Sandalova, Jane H. Buckner, Adnane Achour, Vivianne Malmström

**Affiliations:** ^1^Rheumatology Unit, Department of Medicine Solna, Center for Molecular Medicine, Karolinska Institutet, Karolinska University Hospital, Stockholm, Sweden; ^2^Neuroimmunology Unit, Department of Clinical Neurosciences, Center for Molecular Medicine, Karolinska Institutet, Stockholm, Sweden; ^3^Science for Life Laboratory, Department of Medicine Solna, Karolinska Institutet, Stockholm, Sweden; ^4^Translational Research program, BRI at Virginia Mason, Seattle, WA, USA; ^5^Tetramer Core, BRI at Virginia Mason, Seattle, WA, USA; ^6^Department of Immunology, Genetics and Pathology, Uppsala University, Uppsala, Sweden; ^7^Department of Medical Biochemistry and Biophysics, Karolinska Institutet, Stockholm, Sweden; ^8^Science for Life Laboratory, Department of Medical Sciences, Rheumatology, Uppsala University, Uppsala, Sweden; ^9^Department of Infectious Diseases, Karolinska University Hospital Solna, Stockholm, Sweden

**Keywords:** rheumatoid arthritis, HLA-DR4/α-enolase, neo-antigen, CD4^+^ T cell, autoimmunity, cytokines, crystal structures

The original version of this article contained a typographical error in the spelling of the author Karolina Tandre, which was incorrectly given as Carolina Tandre. The authors apologize for the mistake. This error does not change the scientific conclusions of the article in any way.

The original article was updated.

## Conflict of Interest Statement

The authors declare that the research was conducted in the absence of any commercial or financial relationships that could be construed as a potential conflict of interest.

