# Supplementary material for: Functional and Structural Characterization of a Novel HLA-DRB1*04:01-Restricted α-Enolase T Cell Epitope in Rheumatoid Arthritis
Source: Front Immunol. 2016 Nov 14;7:494. doi: 10.3389/fimmu.2016.00494 (PMC5108039; doi:10.3389/fimmu.2016.00494)
Supplement: Supplementary file 1 [file Presentation_1.PPTX]

## Slide 1
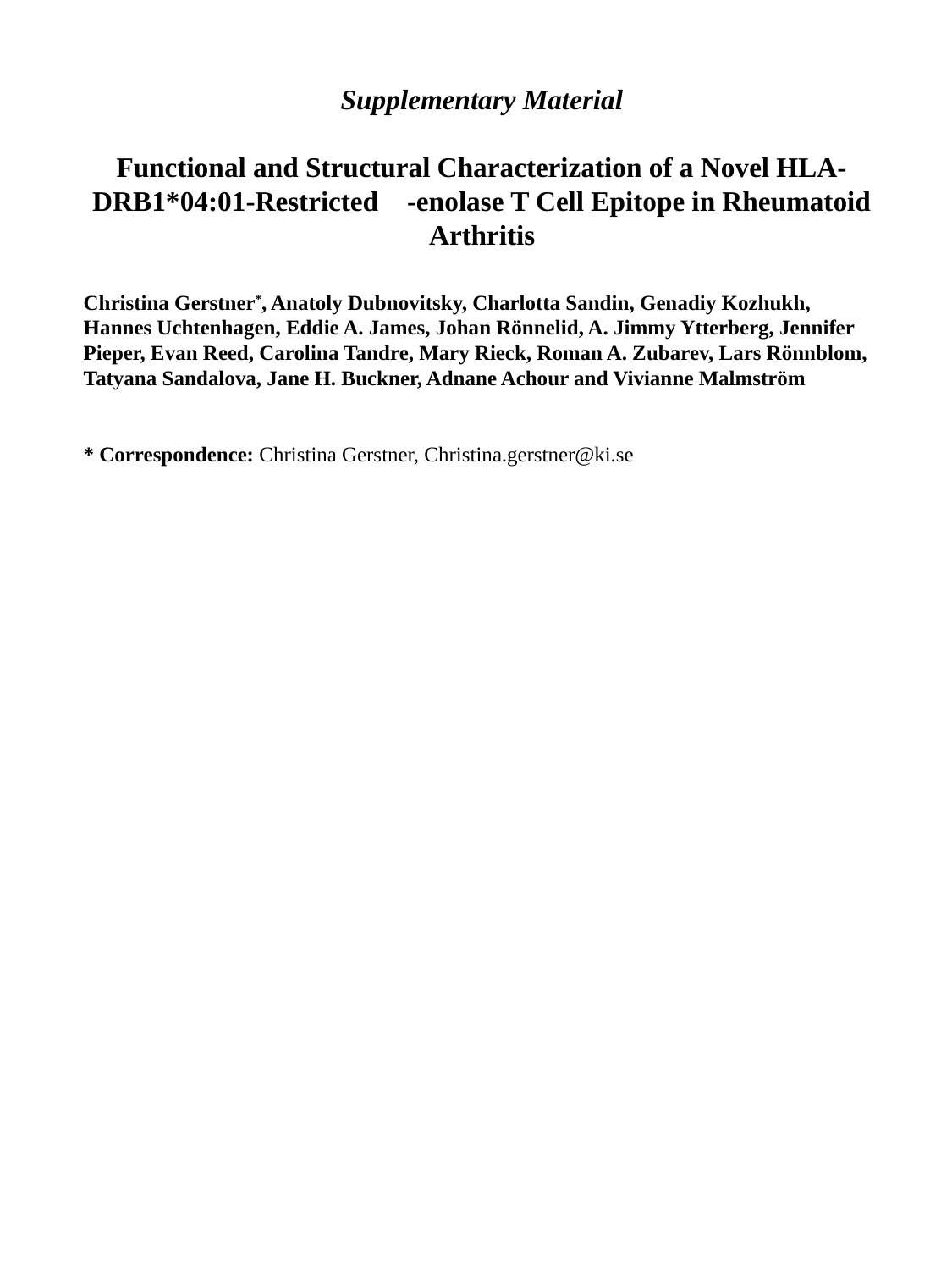

Supplementary Material
Functional and Structural Characterization of a Novel HLA-DRB1*04:01-Restricted α-enolase T Cell Epitope in Rheumatoid Arthritis
Christina Gerstner*, Anatoly Dubnovitsky, Charlotta Sandin, Genadiy Kozhukh, Hannes Uchtenhagen, Eddie A. James, Johan Rönnelid, A. Jimmy Ytterberg, Jennifer Pieper, Evan Reed, Carolina Tandre, Mary Rieck, Roman A. Zubarev, Lars Rönnblom, Tatyana Sandalova, Jane H. Buckner, Adnane Achour and Vivianne Malmström
* Correspondence: Christina Gerstner, Christina.gerstner@ki.se

## Slide 2
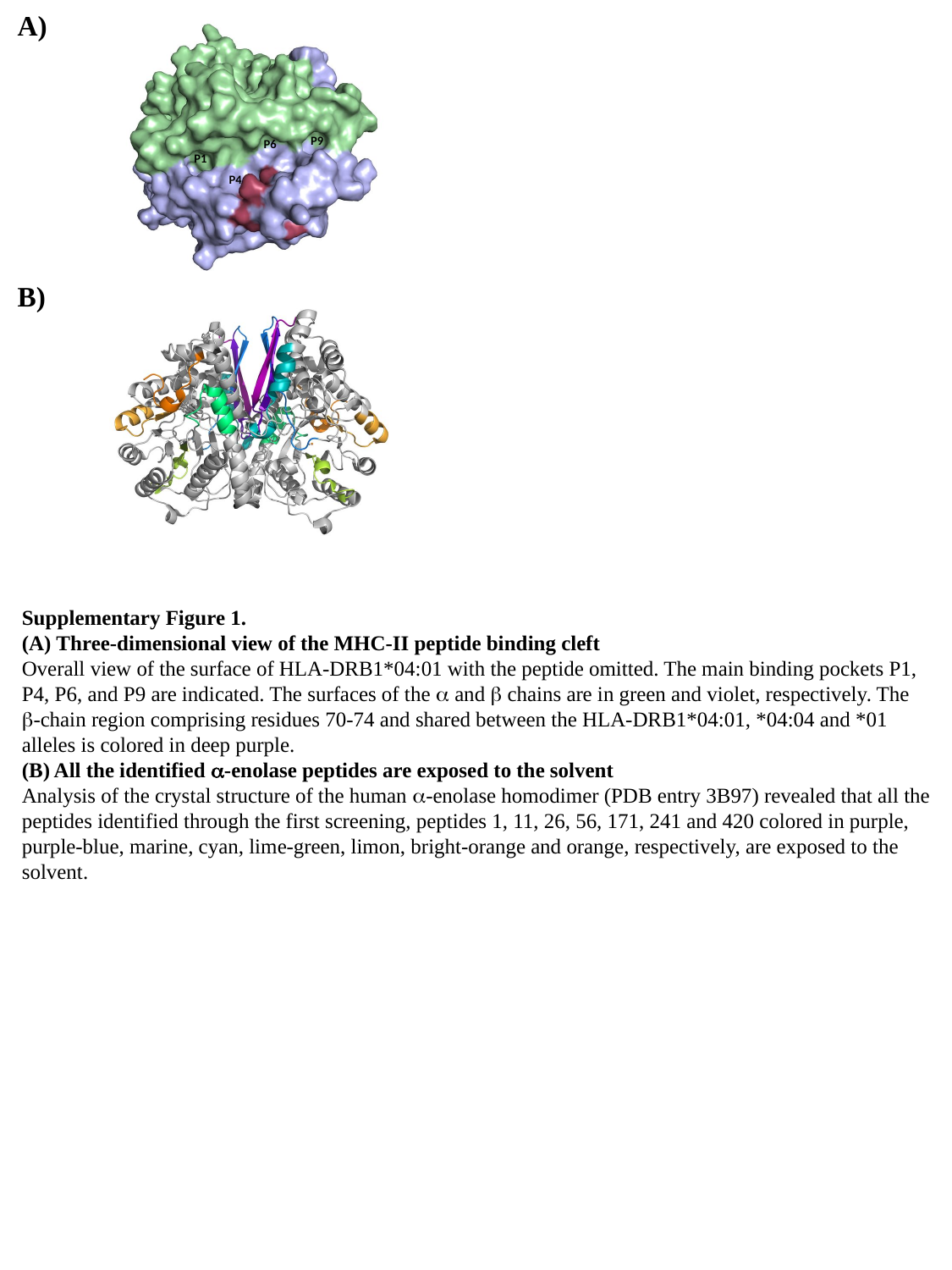

A)
B)
P9
P6
P1
P4
Supplementary Figure 1.
(A) Three-dimensional view of the MHC-II peptide binding cleft
Overall view of the surface of HLA-DRB1*04:01 with the peptide omitted. The main binding pockets P1, P4, P6, and P9 are indicated. The surfaces of the a and b chains are in green and violet, respectively. The b-chain region comprising residues 70-74 and shared between the HLA-DRB1*04:01, *04:04 and *01 alleles is colored in deep purple.
(B) All the identified a-enolase peptides are exposed to the solvent
Analysis of the crystal structure of the human a-enolase homodimer (PDB entry 3B97) revealed that all the peptides identified through the first screening, peptides 1, 11, 26, 56, 171, 241 and 420 colored in purple, purple-blue, marine, cyan, lime-green, limon, bright-orange and orange, respectively, are exposed to the solvent.

## Slide 3
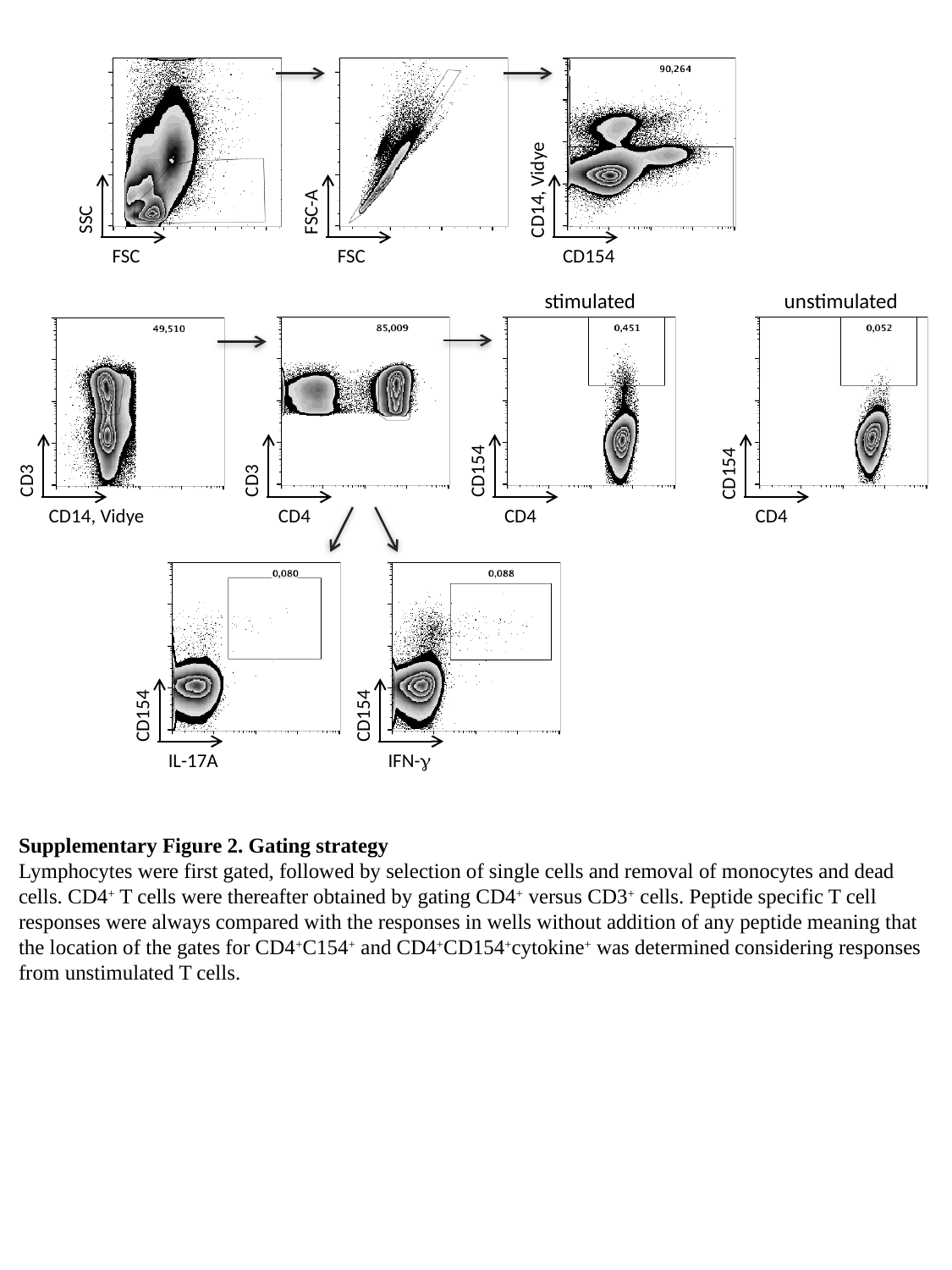

CD14, Vidye
FSC-A
SSC
FSC
FSC
CD154
unstimulated
stimulated
CD154
CD154
CD3
CD3
CD4
CD4
CD4
CD14, Vidye
CD154
CD154
IL-17A
IFN-g
Supplementary Figure 2. Gating strategy
Lymphocytes were first gated, followed by selection of single cells and removal of monocytes and dead cells. CD4+ T cells were thereafter obtained by gating CD4+ versus CD3+ cells. Peptide specific T cell responses were always compared with the responses in wells without addition of any peptide meaning that the location of the gates for CD4+C154+ and CD4+CD154+cytokine+ was determined considering responses from unstimulated T cells.

## Slide 4
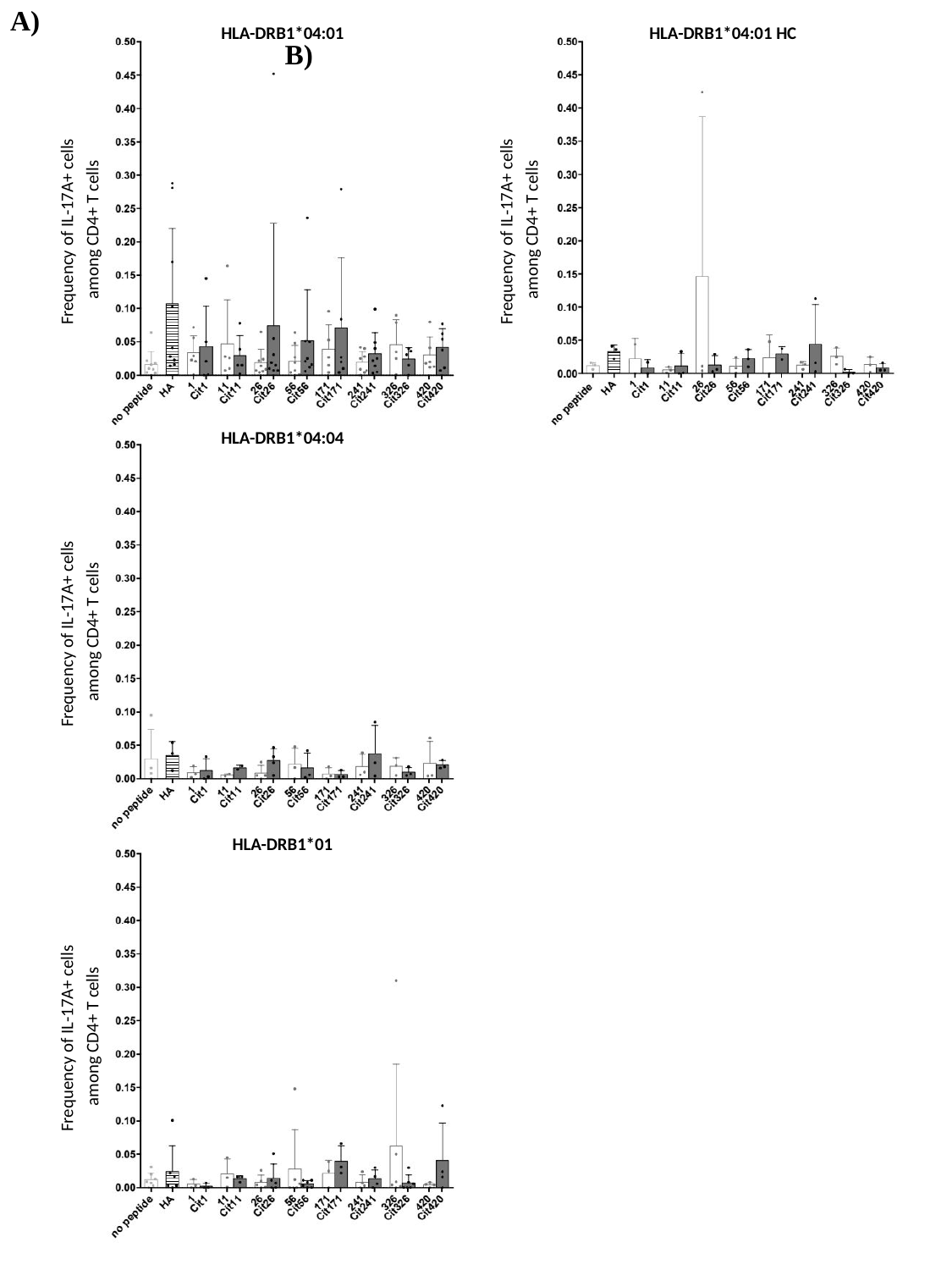

A)							 B)
HLA-DRB1*04:01
HLA-DRB1*04:01 HC
Frequency of IL-17A+ cells
among CD4+ T cells
Frequency of IL-17A+ cells
among CD4+ T cells
HLA-DRB1*04:04
Frequency of IL-17A+ cells
among CD4+ T cells
HLA-DRB1*01
Frequency of IL-17A+ cells
among CD4+ T cells

## Slide 5
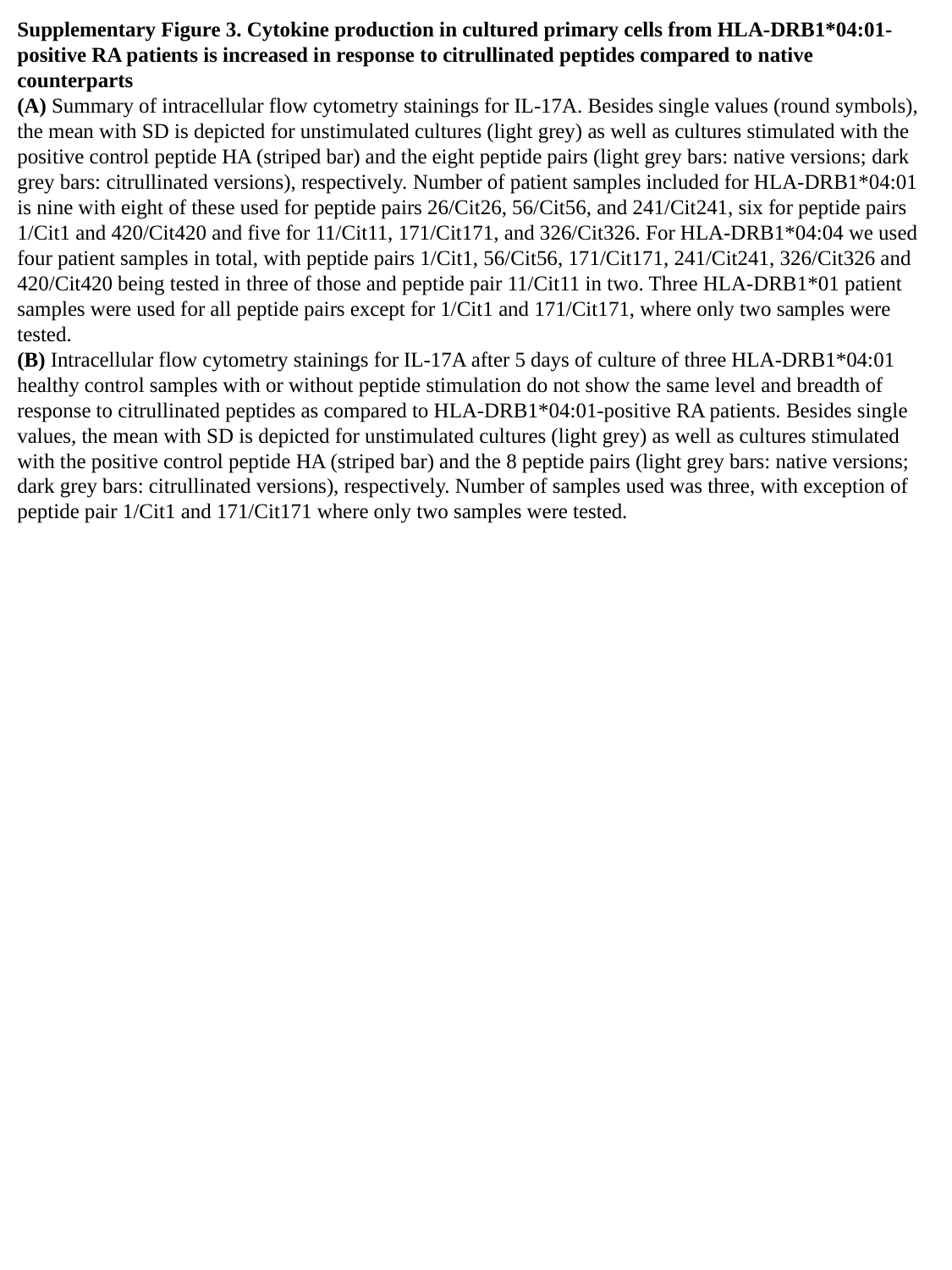

Supplementary Figure 3. Cytokine production in cultured primary cells from HLA-DRB1*04:01-positive RA patients is increased in response to citrullinated peptides compared to native counterparts
(A) Summary of intracellular flow cytometry stainings for IL-17A. Besides single values (round symbols), the mean with SD is depicted for unstimulated cultures (light grey) as well as cultures stimulated with the positive control peptide HA (striped bar) and the eight peptide pairs (light grey bars: native versions; dark grey bars: citrullinated versions), respectively. Number of patient samples included for HLA-DRB1*04:01 is nine with eight of these used for peptide pairs 26/Cit26, 56/Cit56, and 241/Cit241, six for peptide pairs 1/Cit1 and 420/Cit420 and five for 11/Cit11, 171/Cit171, and 326/Cit326. For HLA-DRB1*04:04 we used four patient samples in total, with peptide pairs 1/Cit1, 56/Cit56, 171/Cit171, 241/Cit241, 326/Cit326 and 420/Cit420 being tested in three of those and peptide pair 11/Cit11 in two. Three HLA-DRB1*01 patient samples were used for all peptide pairs except for 1/Cit1 and 171/Cit171, where only two samples were tested.
(B) Intracellular flow cytometry stainings for IL-17A after 5 days of culture of three HLA-DRB1*04:01 healthy control samples with or without peptide stimulation do not show the same level and breadth of response to citrullinated peptides as compared to HLA-DRB1*04:01-positive RA patients. Besides single values, the mean with SD is depicted for unstimulated cultures (light grey) as well as cultures stimulated with the positive control peptide HA (striped bar) and the 8 peptide pairs (light grey bars: native versions; dark grey bars: citrullinated versions), respectively. Number of samples used was three, with exception of peptide pair 1/Cit1 and 171/Cit171 where only two samples were tested.

## Slide 6
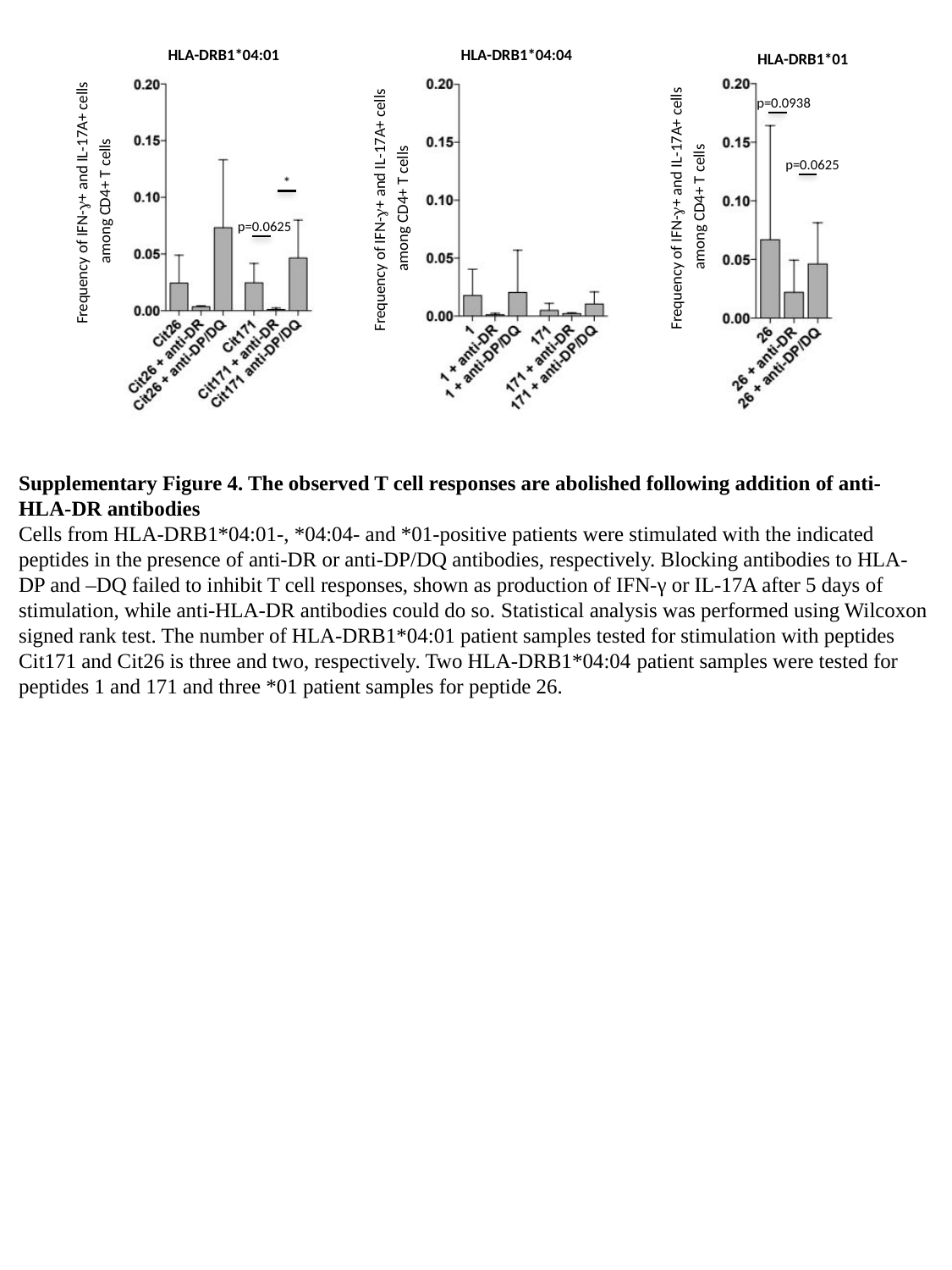

HLA-DRB1*04:01
Frequency of IFN-g+ and IL-17A+ cells
among CD4+ T cells
HLA-DRB1*04:04
Frequency of IFN-g+ and IL-17A+ cells
among CD4+ T cells
HLA-DRB1*01
Frequency of IFN-g+ and IL-17A+ cells
among CD4+ T cells
p=0.0938
p=0.0625
*
p=0.0625
Supplementary Figure 4. The observed T cell responses are abolished following addition of anti-HLA-DR antibodies
Cells from HLA-DRB1*04:01-, *04:04- and *01-positive patients were stimulated with the indicated peptides in the presence of anti-DR or anti-DP/DQ antibodies, respectively. Blocking antibodies to HLA-DP and –DQ failed to inhibit T cell responses, shown as production of IFN-γ or IL-17A after 5 days of stimulation, while anti-HLA-DR antibodies could do so. Statistical analysis was performed using Wilcoxon signed rank test. The number of HLA-DRB1*04:01 patient samples tested for stimulation with peptides Cit171 and Cit26 is three and two, respectively. Two HLA-DRB1*04:04 patient samples were tested for peptides 1 and 171 and three *01 patient samples for peptide 26.

## Slide 7
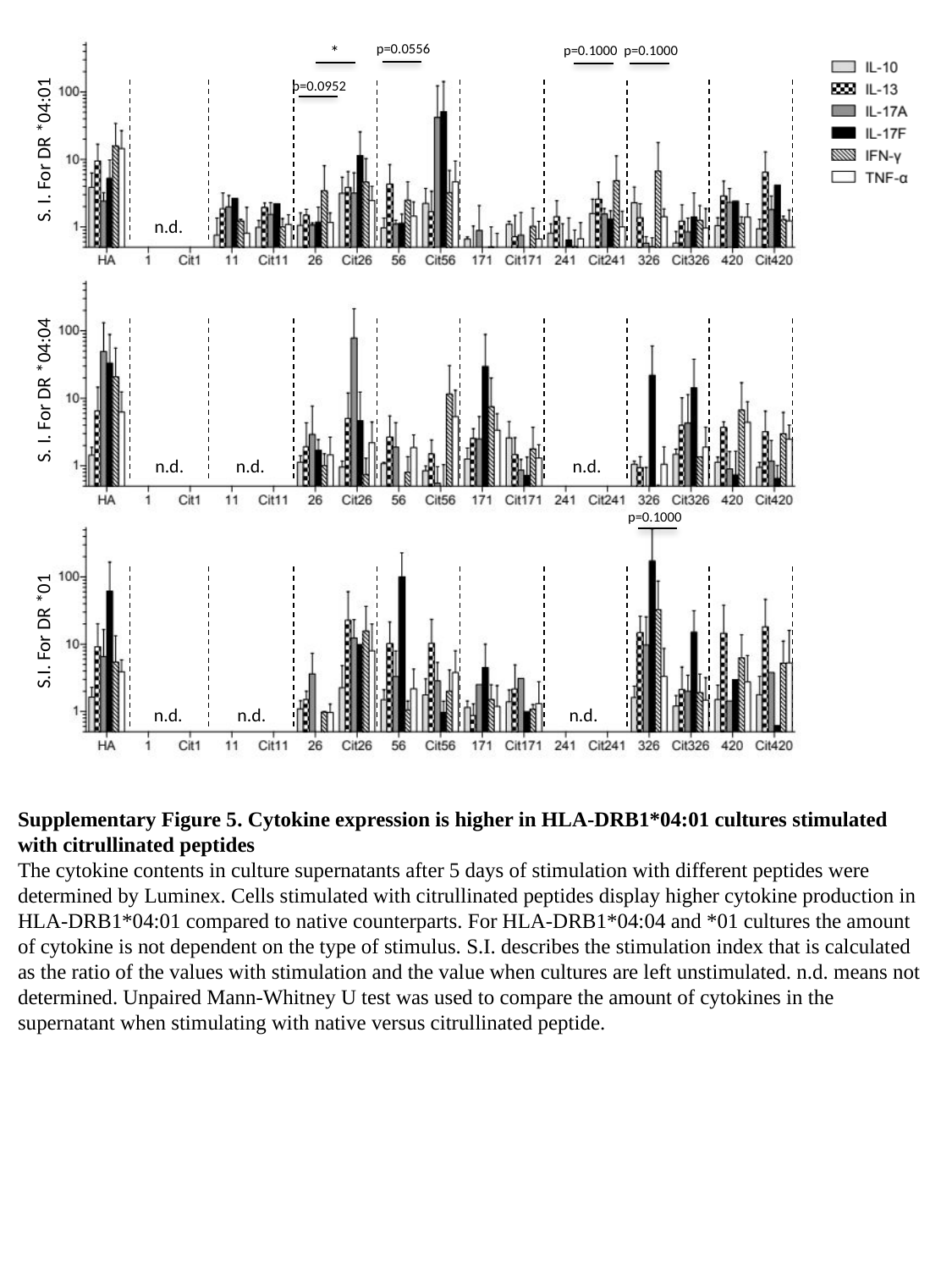

p=0.0556
*
p=0.1000
p=0.1000
p=0.0952
S. I. For DR *04:01
n.d.
S. I. For DR *04:04
n.d.
n.d.
n.d.
p=0.1000
S.I. For DR *01
n.d.
n.d.
n.d.
Supplementary Figure 5. Cytokine expression is higher in HLA-DRB1*04:01 cultures stimulated with citrullinated peptides
The cytokine contents in culture supernatants after 5 days of stimulation with different peptides were determined by Luminex. Cells stimulated with citrullinated peptides display higher cytokine production in HLA-DRB1*04:01 compared to native counterparts. For HLA-DRB1*04:04 and *01 cultures the amount of cytokine is not dependent on the type of stimulus. S.I. describes the stimulation index that is calculated as the ratio of the values with stimulation and the value when cultures are left unstimulated. n.d. means not determined. Unpaired Mann-Whitney U test was used to compare the amount of cytokines in the supernatant when stimulating with native versus citrullinated peptide.

## Slide 8
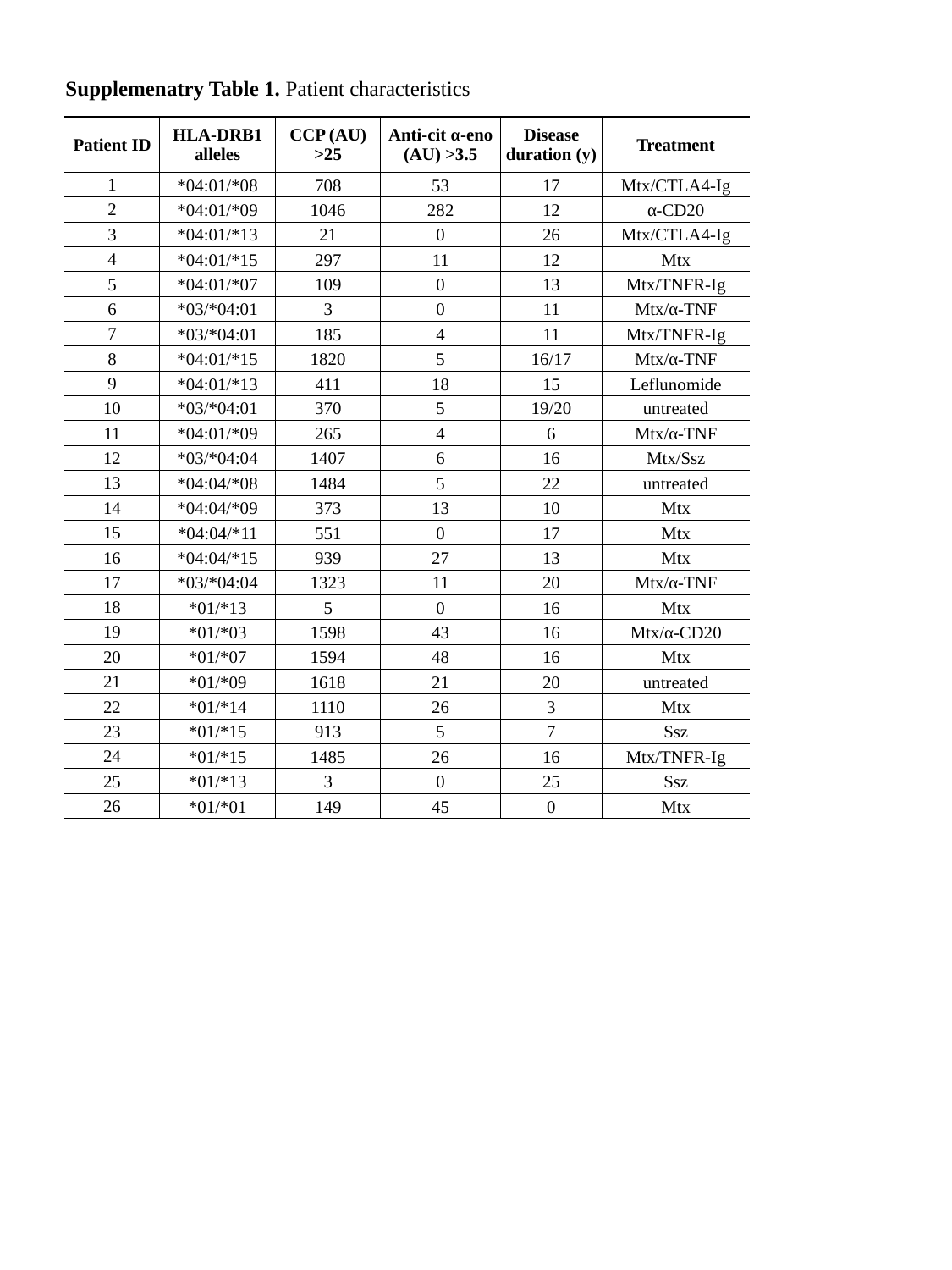

| Supplemenatry Table 1. Patient characteristics | | | | | |
| --- | --- | --- | --- | --- | --- |
| Patient ID | HLA-DRB1 alleles | CCP (AU) >25 | Anti-cit α-eno (AU) >3.5 | Disease duration (y) | Treatment |
| 1 | \*04:01/\*08 | 708 | 53 | 17 | Mtx/CTLA4-Ig |
| 2 | \*04:01/\*09 | 1046 | 282 | 12 | α-CD20 |
| 3 | \*04:01/\*13 | 21 | 0 | 26 | Mtx/CTLA4-Ig |
| 4 | \*04:01/\*15 | 297 | 11 | 12 | Mtx |
| 5 | \*04:01/\*07 | 109 | 0 | 13 | Mtx/TNFR-Ig |
| 6 | \*03/\*04:01 | 3 | 0 | 11 | Mtx/α-TNF |
| 7 | \*03/\*04:01 | 185 | 4 | 11 | Mtx/TNFR-Ig |
| 8 | \*04:01/\*15 | 1820 | 5 | 16/17 | Mtx/α-TNF |
| 9 | \*04:01/\*13 | 411 | 18 | 15 | Leflunomide |
| 10 | \*03/\*04:01 | 370 | 5 | 19/20 | untreated |
| 11 | \*04:01/\*09 | 265 | 4 | 6 | Mtx/α-TNF |
| 12 | \*03/\*04:04 | 1407 | 6 | 16 | Mtx/Ssz |
| 13 | \*04:04/\*08 | 1484 | 5 | 22 | untreated |
| 14 | \*04:04/\*09 | 373 | 13 | 10 | Mtx |
| 15 | \*04:04/\*11 | 551 | 0 | 17 | Mtx |
| 16 | \*04:04/\*15 | 939 | 27 | 13 | Mtx |
| 17 | \*03/\*04:04 | 1323 | 11 | 20 | Mtx/α-TNF |
| 18 | \*01/\*13 | 5 | 0 | 16 | Mtx |
| 19 | \*01/\*03 | 1598 | 43 | 16 | Mtx/α-CD20 |
| 20 | \*01/\*07 | 1594 | 48 | 16 | Mtx |
| 21 | \*01/\*09 | 1618 | 21 | 20 | untreated |
| 22 | \*01/\*14 | 1110 | 26 | 3 | Mtx |
| 23 | \*01/\*15 | 913 | 5 | 7 | Ssz |
| 24 | \*01/\*15 | 1485 | 26 | 16 | Mtx/TNFR-Ig |
| 25 | \*01/\*13 | 3 | 0 | 25 | Ssz |
| 26 | \*01/\*01 | 149 | 45 | 0 | Mtx |

## Slide 9
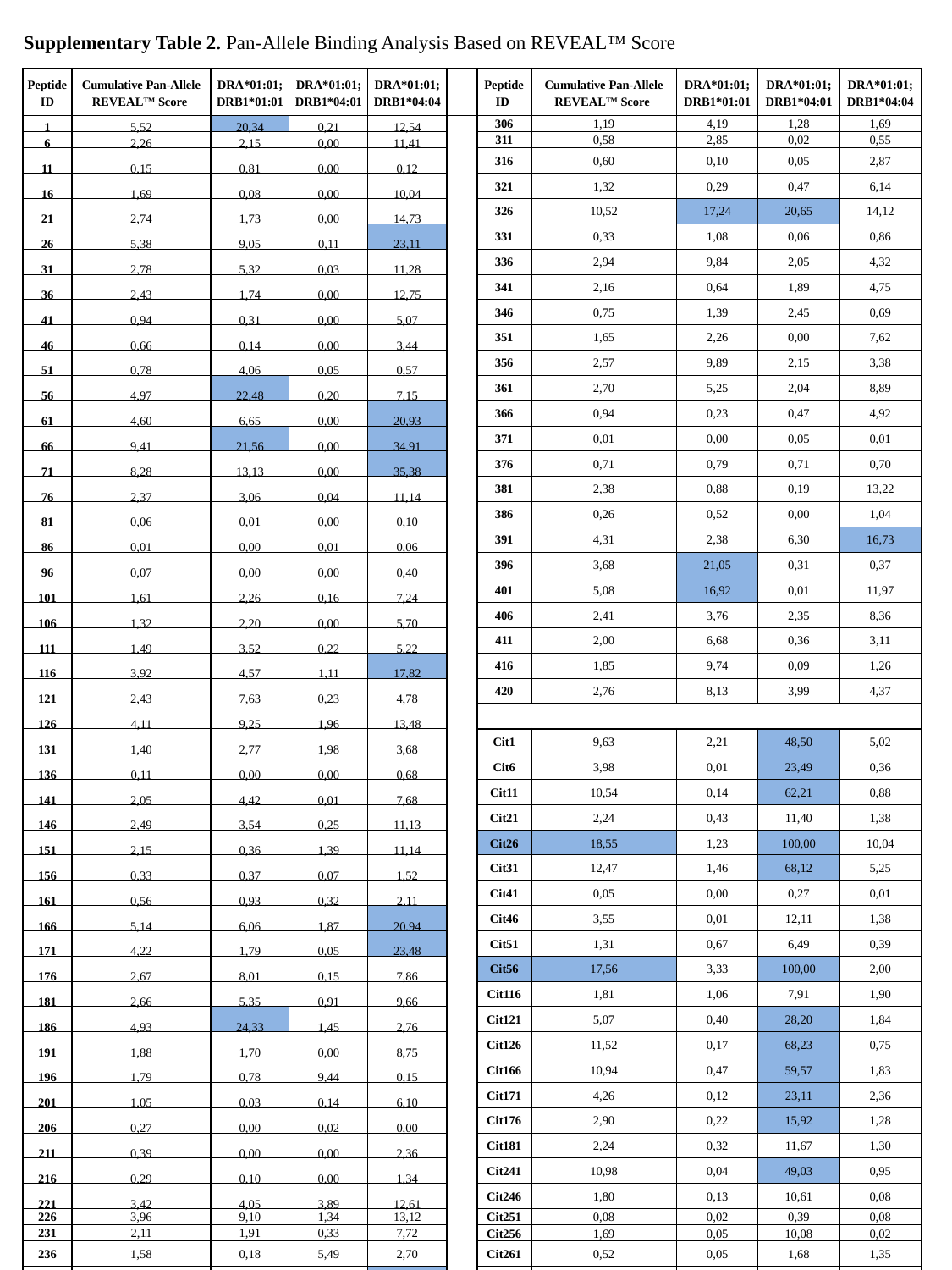

| Supplementary Table 2. Pan-Allele Binding Analysis Based on REVEAL™ Score | | | | | | | | | | |
| --- | --- | --- | --- | --- | --- | --- | --- | --- | --- | --- |
| Peptide ID | Cumulative Pan-Allele REVEALTM Score | DRA\*01:01; DRB1\*01:01 | DRA\*01:01; DRB1\*04:01 | DRA\*01:01; DRB1\*04:04 | | Peptide ID | Cumulative Pan-Allele REVEALTM Score | DRA\*01:01; DRB1\*01:01 | DRA\*01:01; DRB1\*04:01 | DRA\*01:01; DRB1\*04:04 |
| 1 | 5,52 | 20,34 | 0,21 | 12,54 | | 306 | 1,19 | 4,19 | 1,28 | 1,69 |
| 6 | 2,26 | 2,15 | 0,00 | 11,41 | | 311 | 0,58 | 2,85 | 0,02 | 0,55 |
| 11 | 0,15 | 0,81 | 0,00 | 0,12 | | 316 | 0,60 | 0,10 | 0,05 | 2,87 |
| 16 | 1,69 | 0,08 | 0,00 | 10,04 | | 321 | 1,32 | 0,29 | 0,47 | 6,14 |
| 21 | 2,74 | 1,73 | 0,00 | 14,73 | | 326 | 10,52 | 17,24 | 20,65 | 14,12 |
| 26 | 5,38 | 9,05 | 0,11 | 23,11 | | 331 | 0,33 | 1,08 | 0,06 | 0,86 |
| 31 | 2,78 | 5,32 | 0,03 | 11,28 | | 336 | 2,94 | 9,84 | 2,05 | 4,32 |
| 36 | 2,43 | 1,74 | 0,00 | 12,75 | | 341 | 2,16 | 0,64 | 1,89 | 4,75 |
| 41 | 0,94 | 0,31 | 0,00 | 5,07 | | 346 | 0,75 | 1,39 | 2,45 | 0,69 |
| 46 | 0,66 | 0,14 | 0,00 | 3,44 | | 351 | 1,65 | 2,26 | 0,00 | 7,62 |
| 51 | 0,78 | 4,06 | 0,05 | 0,57 | | 356 | 2,57 | 9,89 | 2,15 | 3,38 |
| 56 | 4,97 | 22,48 | 0,20 | 7,15 | | 361 | 2,70 | 5,25 | 2,04 | 8,89 |
| 61 | 4,60 | 6,65 | 0,00 | 20,93 | | 366 | 0,94 | 0,23 | 0,47 | 4,92 |
| 66 | 9,41 | 21,56 | 0,00 | 34,91 | | 371 | 0,01 | 0,00 | 0,05 | 0,01 |
| 71 | 8,28 | 13,13 | 0,00 | 35,38 | | 376 | 0,71 | 0,79 | 0,71 | 0,70 |
| 76 | 2,37 | 3,06 | 0,04 | 11,14 | | 381 | 2,38 | 0,88 | 0,19 | 13,22 |
| 81 | 0,06 | 0,01 | 0,00 | 0,10 | | 386 | 0,26 | 0,52 | 0,00 | 1,04 |
| 86 | 0,01 | 0,00 | 0,01 | 0,06 | | 391 | 4,31 | 2,38 | 6,30 | 16,73 |
| 96 | 0,07 | 0,00 | 0,00 | 0,40 | | 396 | 3,68 | 21,05 | 0,31 | 0,37 |
| 101 | 1,61 | 2,26 | 0,16 | 7,24 | | 401 | 5,08 | 16,92 | 0,01 | 11,97 |
| 106 | 1,32 | 2,20 | 0,00 | 5,70 | | 406 | 2,41 | 3,76 | 2,35 | 8,36 |
| 111 | 1,49 | 3,52 | 0,22 | 5,22 | | 411 | 2,00 | 6,68 | 0,36 | 3,11 |
| 116 | 3,92 | 4,57 | 1,11 | 17,82 | | 416 | 1,85 | 9,74 | 0,09 | 1,26 |
| 121 | 2,43 | 7,63 | 0,23 | 4,78 | | 420 | 2,76 | 8,13 | 3,99 | 4,37 |
| 126 | 4,11 | 9,25 | 1,96 | 13,48 | | | | | | |
| 131 | 1,40 | 2,77 | 1,98 | 3,68 | | Cit1 | 9,63 | 2,21 | 48,50 | 5,02 |
| 136 | 0,11 | 0,00 | 0,00 | 0,68 | | Cit6 | 3,98 | 0,01 | 23,49 | 0,36 |
| 141 | 2,05 | 4,42 | 0,01 | 7,68 | | Cit11 | 10,54 | 0,14 | 62,21 | 0,88 |
| 146 | 2,49 | 3,54 | 0,25 | 11,13 | | Cit21 | 2,24 | 0,43 | 11,40 | 1,38 |
| 151 | 2,15 | 0,36 | 1,39 | 11,14 | | Cit26 | 18,55 | 1,23 | 100,00 | 10,04 |
| 156 | 0,33 | 0,37 | 0,07 | 1,52 | | Cit31 | 12,47 | 1,46 | 68,12 | 5,25 |
| 161 | 0,56 | 0,93 | 0,32 | 2,11 | | Cit41 | 0,05 | 0,00 | 0,27 | 0,01 |
| 166 | 5,14 | 6,06 | 1,87 | 20,94 | | Cit46 | 3,55 | 0,01 | 12,11 | 1,38 |
| 171 | 4,22 | 1,79 | 0,05 | 23,48 | | Cit51 | 1,31 | 0,67 | 6,49 | 0,39 |
| 176 | 2,67 | 8,01 | 0,15 | 7,86 | | Cit56 | 17,56 | 3,33 | 100,00 | 2,00 |
| 181 | 2,66 | 5,35 | 0,91 | 9,66 | | Cit116 | 1,81 | 1,06 | 7,91 | 1,90 |
| 186 | 4,93 | 24,33 | 1,45 | 2,76 | | Cit121 | 5,07 | 0,40 | 28,20 | 1,84 |
| 191 | 1,88 | 1,70 | 0,00 | 8,75 | | Cit126 | 11,52 | 0,17 | 68,23 | 0,75 |
| 196 | 1,79 | 0,78 | 9,44 | 0,15 | | Cit166 | 10,94 | 0,47 | 59,57 | 1,83 |
| 201 | 1,05 | 0,03 | 0,14 | 6,10 | | Cit171 | 4,26 | 0,12 | 23,11 | 2,36 |
| 206 | 0,27 | 0,00 | 0,02 | 0,00 | | Cit176 | 2,90 | 0,22 | 15,92 | 1,28 |
| 211 | 0,39 | 0,00 | 0,00 | 2,36 | | Cit181 | 2,24 | 0,32 | 11,67 | 1,30 |
| 216 | 0,29 | 0,10 | 0,00 | 1,34 | | Cit241 | 10,98 | 0,04 | 49,03 | 0,95 |
| 221 | 3,42 | 4,05 | 3,89 | 12,61 | | Cit246 | 1,80 | 0,13 | 10,61 | 0,08 |
| 226 | 3,96 | 9,10 | 1,34 | 13,12 | | Cit251 | 0,08 | 0,02 | 0,39 | 0,08 |
| 231 | 2,11 | 1,91 | 0,33 | 7,72 | | Cit256 | 1,69 | 0,05 | 10,08 | 0,02 |
| 236 | 1,58 | 0,18 | 5,49 | 2,70 | | Cit261 | 0,52 | 0,05 | 1,68 | 1,35 |
| 241 | 5,53 | 2,35 | 9,89 | 16,80 | | Cit266 | 2,49 | 0,45 | 11,82 | 2,64 |
| 246 | 0,23 | 0,32 | 0,97 | 0,12 | | Cit316 | 0,67 | 0,01 | 2,47 | 1,40 |
| 251 | 0,93 | 0,31 | 0,04 | 0,03 | | Cit321 | 1,14 | 0,69 | 3,01 | 3,13 |
| 256 | 0,77 | 0,00 | 0,00 | 4,59 | | Cit326 | 25,35 | 5,99 | 100,00 | 6,49 |
| 261 | 0,94 | 2,77 | 0,07 | 2,70 | | Cit361 | 3,21 | 0,06 | 17,65 | 1,55 |
| 266 | 0,07 | 0,16 | 0,10 | 0,18 | | Cit366 | 0,48 | 0,00 | 2,22 | 0,63 |
| 271 | 0,29 | 0,00 | 0,00 | 1,52 | | Cit391 | 8,50 | 0,00 | 48,75 | 2,24 |
| 276 | 1,71 | 2,50 | 0,86 | 5,01 | | Cit396 | 0,51 | 0,43 | 2,63 | 0,00 |
| 281 | 9,04 | 20,40 | 15,83 | 9,09 | | Cit401 | 0,09 | 0,13 | 0,05 | 0,38 |
| 286 | 1,10 | 0,00 | 0,02 | 3,35 | | Cit406 | 2,15 | 0,35 | 11,69 | 0,87 |
| 291 | 0,01 | 0,00 | 0,07 | 0,00 | | Cit411 | 1,05 | 0,49 | 4,38 | 0,59 |
| 296 | 0,02 | 0,00 | 0,09 | 0,00 | | Cit416 | 1,76 | 0,36 | 8,09 | 2,13 |
| 301 | 1,70 | 7,93 | 0,74 | 1,52 | | Cit420 | 9,67 | 0,79 | 53,04 | 4,20 |
| The REVEAL™ binding score for each peptide-MHC complex is calculated at 0 and 24 h by comparison to the binding of the relevant positive control at 0 h and tabulated numerically. Tabulated representation of REVEAL™ scores for HLA-DRA\*01:01 in complex with HLA-DRB1\*01:01, \*04:01, \*04:04 are presented. Samples with REVEAL™ binding assay scores >15% are highlighted in blue. | | | | | | | | | | |
